# Literature-based meta-analysis of adverse events accounting for heterogeneous follow-up duration in oncology clinical trials

**DOI:** 10.1017/rsm.2026.10083

**Published:** 2026-04-08

**Authors:** Sumika Kawaguchi, Satoshi Hattori

**Affiliations:** 1 Clinical Study Support Center, Wakayama Medical University Hospital, Japan; 2 Department of Biomedical Statistics, Graduate School of Medicine and Institute for Open and Transdisciplinary Research Initiatives (OTRI), The University of Osaka, Japan

**Keywords:** copula, graph scan software, Kaplan–Meier estimator, rare events, survival analysis

## Abstract

It is difficult to understand the safety profile of drugs based on a single clinical trial since clinical trials are often designed to prove efficacies, and sample size is not powered for safety assessment. Thus, meta-analysis would be a valuable tool to infer the safety profiles utilizing multiple studies. Individual clinical trials usually report the incidence proportions of adverse events (AEs) observed in the study. The follow-up duration may be study-specific, and furthermore different between the treatment groups within a single study. It often occurs in oncology clinical trials and if this is the case, it is hard to interpret the aggregated relative risk of AEs and compare the risk of AEs between the treatment groups with the standard meta-analysis techniques. The progression-free survival or the overall survival is often used as the primary endpoint in oncology clinical trials and the Kaplan–Meier estimates of the survival functions for the primary endpoint are often demonstrated graphically, which give us information of the follow-up duration of the AEs. We propose novel meta-analysis methods for AEs that address differences in follow-up durations by efficiently utilizing the Kaplan–Meier estimates of the primary endpoint. We adapt our approach using both simulated data and real data from a meta-analysis of bevacizumab. Simulation studies demonstrate that the proposed methods perform well when follow-up time differs between trials and groups.

## Highlights

### What is already known?

- Incidence proportions are often used in meta-analyses of adverse events.
- Heterogeneity in follow-up duration often occurs in meta-analyses of adverse events in randomized clinical trials, particularly in oncology, and this makes it difficult to interpret pooled incidence proportions using standard meta-analytic methods.
- Although survival analysis techniques would be useful for accounting for heterogeneous follow-up durations, individual studies rarely report summary measures based on survival analysis.
- No commonly accepted methods are available for literature-based meta-analyses of adverse events.

### What is new?

- Novel methods are proposed to estimate cumulative incidence functions for adverse events using incidence proportions reported in individual studies, while accounting for heterogeneous follow-up durations.

### Potential impact for RSM readers

- The proposed method enables fair comparisons of adverse event occurrence by appropriately adjusting for heterogeneity in follow-up duration across treatments.
- This approach would be beneficial for evaluating the risk–benefit balance of treatments.

## 1 Introduction

In clinical trials, it is often of primary interest to investigate efficacies of some drugs. Even if the primary endpoint is an efficacy parameter, safety assessment is a crucial aspect in all clinical trials. Details of all the adverse events (AEs) and their frequencies observed in the trial should be reported. For each AE, the incidence proportion, which is the number of subjects who experienced the AE divided by the number of subjects, is usually reported. Most randomized clinical trials are primarily designed to compare efficacies. Unless a randomized controlled clinical trial is prospectively designed with a specific safety outcome as its primary endpoint and sized accordingly, the trial may lack sufficient sample size to reliably assess whether there is an increased risk of AEs. In other words, a single clinical trial may have limited capacity to evaluate drug safety and then synthesizing information of AEs over multiple studies would provide valuable information.

Thus, meta-analysis would offer a valuable framework to understand safety profiles of drugs. In meta-analysis of AEs, the incidence proportion is often of primary interest, and the empirical incidence proportions are aggregated over studies and compared across treatment groups. For example, Zhu et al.^1^ conducted a meta-analysis to examine whether bevacizumab increased cerebrovascular event risks. Cortes et al.^2^ reported a meta-analysis of the AEs for breast cancer patients treated by bevacizumab and Gyawail et al.^3^ investigated serious and fatal AE risks of sorafenib with solid tumors. In the standard meta-analysis, the fixed-effect and the mixed-effect models are often used; the former assumes that the treatment effect is common, and the latter allows for heterogeneity over studies. To estimate the overall treatment effect, the inverse variance weighted average estimator, the normal–normal model, and the generalized linear mixed model are often used. In general, the incidence proportions of AEs are likely to be very low and may be zero for some studies. Thus, the standard methods for meta-analysis may not work in such situations with sparse data and several alternative methods have been developed taking account for such sparsity of the frequencies.^4^ ^–^ ^6^

In addition to the sparsity issue, various issues specific to meta-analysis of AEs were discussed by a regulatory guidance document^7^ and Cochrane Handbook (see Chapter 19 of Higgins and Thomas^8^). Heterogeneity in follow-up duration for evaluation of AEs is one of them (see Section III-D of FDA guidance^7^). The duration of follow-up may differ among the treatment groups and thus should be factored into safety meta-analysis. Varying follow-up durations between the treatment groups due to discontinuing assigned drugs or dropouts may occur and impact the comparability among groups with respect to safety outcomes. If this is the case, aggregating the incidence proportions may be misleading and less informative.

When subject-level data are available, survival analysis techniques can be employed to account for differences in follow-up duration among studies. Stegherr et al.^9^ ^,^ ^10^ recommended use of survival analysis techniques in the analysis of AEs in clinical trials. However, such meticulous analyses are seldom executed in individual trials, and conducting individual participants data (IPD) meta-analysis is also challenging with difficulty to collect all the IPDs. Consequently, meta-analysis based on study-level data (literature-based meta-analysis) necessitates approaches that can accommodate disparities in follow-up duration. To our best knowledge, no formal methods have been proposed to handle heterogeneous follow-up duration in aggregated meta-analysis.

In clinical trials in oncology, the primary endpoint is often a time-to-event endpoint, such as the overall survival (OS) and the progression-free-survival (PFS). If the primary endpoint is the OS, the incidence proportion of the AEs is only for the period from the date of study initiation (typically the date of randomization) to the OS. Then, if the OSs are substantially longer in the experimental treatment than in the control treatment, it may be misleading to compare the incidence proportions between the treatment groups. This happens even with the PFS as the primary endpoint. Ethical care for subjects often leads to the treatment discontinuation and the subject is off the study once disease progression is observed. More exactly, in practice, with an extra follow-up duration of AEs such as one month, AEs are followed up to certain period after the PFS (e.g., PFS+1 month). If this is the case, interpreting the incidence proportion becomes difficult. Note that the follow-up scheme on the PFS is usually defined in the study protocol in oncology clinical trials. Thus, the issue of the heterogeneous follow-up duration is likely to arise common in oncology trials.

On the other hand, the statistical analysis for the time-to-event primary endpoint (e.g., PFS and OS) are usually conducted with the Kaplan–Meier method and since showing benefits in efficacy is of primary interest, the Kaplan–Meier estimates of the survival functions of the treatment groups are highlighted graphically in almost all the oncology studies. Kaplan–Meier estimates can provide valuable information regarding follow-up durations for AEs. In this article, we propose novel safety meta-analysis methods that address difference in follow-up duration for AEs among the treatment groups. Our idea is to analyze the AE as a time-to-event outcome by considering the cumulative incidence function as the estimand and propose estimation procedures for it utilizing the Kaplan–Meier estimates of the time-to-event primary endpoint, which form the distribution of the follow-up duration of AEs. At first, we introduce our proposed method assuming that the follow-up duration (e.g., PFS and OS) is independent of the time-to-AE. In practice, this independence assumption may be questionable. Thus, we also introduce an inference procedure accounting for dependence between the time-to-AE and the follow-up duration with copulas. As far as we know, our approach is the first safety meta-analysis method that can address difference in the follow-up duration.

The remainder of this article is structured as follows. Section 2 provides a motivating example of the meta-analysis of AEs in randomized clinical trials for bevacizumab and introduce the setting and notations. In Section 3, we introduce our proposed methods. We illustrate our proposed method by re-analyzing the meta-analysis of bevacizumab in Section 4 and examine the performance of the method by simulation studies in Section 5. Finally, we conclude this manuscript by describing advantages and limitations of the proposed method and potential future work in Section 6.

## 2 Preliminary

### 2.1 Motivating example: Meta-analysis of adverse events with bevacizumab

Our study was motivated by a meta-analysis of cerebrovascular events of anti-neoplastic agent bevacizumab.^1^ This meta-analysis included 12,917 patients from 17 randomized clinical trials and the primary interest was to evaluate cerebrovascular events when administrated by bevacizumab in cancer patients. Basic characteristics of the studies are summarized in Table 1 of Zhu et al.^1^ Studies of various cancer types were included with bevacizumab as an experimental treatment group. The primary outcome measure of the meta-analysis was the risk ratio (RR) of the bevacizumab group to the control, which was calculated as the ratio of the incidence proportion for occurrence of cerebrovascular events of the bevacizumab group to that of the control treatment. The RRs of the incidence proportion were aggregated by using the Mantel–Haenszel method over the studies. They reported the aggregated RR 3.28 (95%CI:1.97–5.48), concluding that bevacizumab had a significantly higher cerebrovascular events risk.

**Table 1:** Summary of availability of information required by the proposed method. *n*: the number of subjects for safety analysis; MFT: median follow-up time (month); IP: incidence proportion (bev.: bevacizumab group and cnt.: control group); KM: availability of Kaplan–Meier estimates of the primary endpoint (open circle: reported and cross: missing); Follow-up: definition of the end of-follow-up for AEs in the protocol (Month); At-risk: availability of at-risk information for the primary endpoint typically attached below the figure for Kaplan–Meier estimates (open circle: reported and cross: missing); and NA: not available Table 1 Long description.

No.	Study ID	*n*	MFT(Mo)	IP (bev.)	IP (cnt.)	Primary endpoint	Follow-up	KM	At-risk
1	Aghajanian, 2012	480	24	2/247=0.81%	1/233=0.43%	PFS	PFS+1 (Mo)	◯	◯
2	Allegra, 2009	2,647	28.5	5/1326=0.38%	5/1321=0.38%	AE incidence	NA	×	×
3	Burger 2011	1,209	17.4	2/608=0.33%	0/601=0.00%	PFS	PFS+1 (Mo)	◯	◯
4	Chinot 2014	921	NA	9/461=1.95%	4/450=0.89%	OS, PFS	PFS+3 (Mo)	◯	◯
5	Giantonio, 2007	572	28	3/287=1.04%	0/285=0.00%	OS	OS	◯	×
6	Herbst, 2011	626	19	1/313=0.32%	0/313=0.00%	OS	OS	◯	◯
7	Kabbinavar, 2003	102	17.6	2/32=6.25%	0/35=0.00%	PFS	NA	×	×
8	Kelly, 2012	1,009	25	7/504=1.39%	1/505=0.20%	OS	PFS+1 (Mo)	◯	◯
9	Kim, 2012	212	13	0/214=0.00%	0/212=0.00%	PFS	PFS+1 (Mo)	◯	◯
10	Kindler, 2007	108	15.6	2/53=3.77%	0/53=0.00%	PFS	NA	×	×
11	Kindler, 2010	540	NA	2/277=0.72%	2/263=0.76%	OS	OS	◯	×
12	Miller, 2007	711	41.6	7/365=1.92%	0/346=0.00%	PFS	OS	◯	◯
13	Okines, 2013	200	22	1/99=1.01%	0/101=0.00%	GI perforation rates	NA	×	×
14	Perren, 2011	1,528	NA	2/745=0.27%	0/753=0.00%	OS, PFS	PFS+1.5 (Mo)	◯	◯
15	Price, 2012	471	30.8	4/315=1.27%	0/236=0.00%	OS, PFS	NA	×	×
16	Rini, 2010	709	NA	5/362=1.38%	1/347=0.29%	OS	OS	◯	◯
17	Sandler, 2006	867	19	5/427=1.17%	0/440=0.00%	OS	OS	◯	×

Table 1 of the present article summarizes some additional characteristics of the enrolled studies, as well as important information from Table 1 of Zhu et al.^1^ Among the 17 studies, 15 studies were designed to demonstrate efficacy of bevacizumab for a time-to-event endpoint. We searched descriptions on the definition of the end of follow-up in each individual publication. As presented in Table 1, some studies with the PFS as the primary endpoint discontinued the study follow-up after certain duration such as one month after the PFS was determined. In many studies, efficacy of bevacizumab was established with statistical significance. For example, Aghajanian et al.,^11^ which is the study 1 in Table 1, conducted a doube blind, randomized, and placebo-controlled clinical trial for endometrial cancer patients of bevacizumab. The primary endpoint was the PFS and they reported a statistically significant prolongation of the PFS with bevacizumab; the median PFS was 12.4 month in bevacizumab group and that in the control was 8.4 month. In the study protocol of Aghajanian et al.,^11^ the study was designed to discontinue follow-up for a patient at one month after a progression event. Therefore, the follow-up duration of the AE was longer in the bevacizumab group than the control group. Aghajanian et al.^11^ reported the incidence proportion of the bevacizumab group as 2/247=0.008 and that of the control group as 1/233=0.004. Since the event rates were very low, it was hard to compare these two incidence proportions. Furthermore, heterogeneous follow-up durations between the groups complicated the comparison. Aggregation over studies may solve the issue of rare events, but cannot solve the heterogeneous follow-up issue. Then, it is unclear what the aggregated RR 3.28^1^ estimated.

### 2.2 Setting and notation

Suppose we are interested in conducting a meta-analysis of AEs for randomized clinical trials in oncology. We consider a literature-based meta-analysis based on summary statistics (not IPD) reported in individual studies. Let the number of studies in the meta-analysis be denoted by *S*. Each individual study was designed to compare a time-to-event efficacy endpoint, such as the OS and the PFS between two treatment groups (experimental/control). Let *k*= 1 imply the experimental treatment and *k*=0 the control group. The number of subjects in the *k*th group of the *s*th study is denoted by $n_{k}^{\left(s\right)}$ . To describe our proposed method, we need to prepare many time-to-event variables for individual participants, corresponding cumulative distribution functions and survival functions, and observed data. In Table 2, we summarize key notations. We begin with preparing notations for individuals in each study, which are not observed in the literature-based meta-analysis. As introduced in Section 2.1, the follow-up is discontinued once the primary time-to-event endpoint is confirmed; for the OS, the follow-up is until the date to die and for the PFS, after some extra duration defined in the study protocol, say one month, from the PFS, the follow-up is discontinued due to ethical consideration. We consider several random variables for time-to-events. We set the date of randomization as the origin of them. Let the time until the primary endpoint of the *i*th subject of the *k*th group in the *s*th study be denoted by $T_{k,i}^{\left(s\right)}$ and it is right-censored by $C_{k,i}^{\left(s\right)}$ , which is the end of the follow-up of the efficacy endpoint. Let ${\tilde{T}}_{k,i}^{\left(s\right)}=min(T_{k,i}^{\left(s\right)},C_{k,i}^{\left(s\right)})$ and $\Delta_{k,i}^{\left(s\right)}=I(T_{k,i}^{\left(s\right)}\leq C_{k,i}^{\left(s\right)})$ . The pair $\left({\tilde{T}}_{k,i}^{\left(s\right)},\Delta_{k,i}^{\left(s\right)}\right)$ is the standard data for survival analysis. We suppose the standard assumption for survival analysis; $T_{k,i}^{\left(s\right)}$ and $C_{k,i}^{\left(s\right)}$ are independent, which ensures validity of the Kaplan–Meier estimator. Let the survival and cumulative distribution functions of $T_{k,i}^{\left(s\right)}$ be denoted by $S_{k}^{\left(s\right)}(t)=P(T_{k,i}^{\left(s\right)}>t)$ and $F_{k}^{\left(s\right)}(t)=1-S_{k}^{\left(s\right)}(t)$ , respectively. The Kaplan–Meier estimate for $S_{k}^{\left(s\right)}(t)$ based on the observations $\left({\tilde{T}}_{k,i}^{\left(s\right)},\Delta_{k,i}^{\left(s\right)}\right)$ ( $i=1,2,\ldots ,n_{k}^{\left(s\right)}$ ) is denoted by ${\hat{S}}_{k}^{\left(s\right)}(t)$ . Denote $G_{k}^{\left(s\right)}(t)=P(C_{k,i}^{\left(s\right)}>t)$ . Let the time until the end of follow-up be denoted by $T_{FU:k,i}^{\left(s\right)}$ ; for the OS, $T_{FU:k,i}^{\left(s\right)}$ agrees with $T_{k,i}^{\left(s\right)}$ and for the PFS, extra follow-up duration, say one month, is added according to the procedure defined in the study protocol. The survival and cumulative distribution functions for $T_{FU:k,i}^{\left(s\right)}$ are denoted by $S_{FU:k}^{\left(s\right)}(t)$ and $F_{FU:k}^{\left(s\right)}(t)$ , respectively. Let ${\tilde{T}}_{FU:k,i}^{\left(s\right)}=min(T_{FU:k,i}^{\left(s\right)},C_{k,i}^{\left(s\right)})$ and its survival and cumulative distribution functions be denoted by ${\tilde{S}}_{FU:k}^{\left(s\right)}(t)$ and ${\tilde{F}}_{FU:k}^{\left(s\right)}(t)$ , respectively. Furthermore, we define the time-to-AE, $T_{AE:k,i}^{\left(s\right)}$ by the duration of the day of the first occurrence of the AE from the day of randomization. We assume that the distribution of $T_{AE:k,i}^{\left(s\right)}$ is common over the studies, and then denote its cumulative distribution function and its survival function by $F_{AE:k}(t)$ and $S_{AE:k}(t)$ , respectively, dropping the superscript for dependence to the study.

**Table 2:** List of notations Table 2 Long description.

Index	s: study ( s=1,2,…,S )
	*k*: treatment group (1: experimental, 0: control)
	*i*: subject
Time-to-event variable	$T_{k,i}^{\left(s\right)}$ : time to primary endpoint (OS or PFS)
	$C_{k,i}^{\left(s\right)}$ : potential censoring time for $T_{k,i}^{\left(s\right)}$
	${\tilde{T}}_{k,i}^{\left(s\right)}=min(T_{k,i}^{\left(s\right)},C_{k,i}^{\left(s\right)})$
	$T_{AE:k,i}^{\left(s\right)}$ : time to AE
	$T_{FU:k,i}^{\left(s\right)}$ : follow-up duration for AE
	${\tilde{T}}_{FU:k,i}^{\left(s\right)}=min(T_{FU:k,i}^{\left(s\right)},C_{k,i}^{\left(s\right)})$
Function	$S_{k}^{\left(s\right)}(t)=P(T_{k,i}^{\left(s\right)}>t)$
	$F_{k}^{\left(s\right)}(t)=1-S_{k}^{\left(s\right)}(t)$
	$G_{k}^{\left(s\right)}(t)=P(C_{k,i}^{\left(s\right)}>t)$
	$S_{FU:k}^{\left(s\right)}(t)=P(T_{FU:k,i}^{\left(s\right)}>t)$
	$F_{FU:k}^{\left(s\right)}(t)=1-S_{FU:k}^{\left(s\right)}(t)$
	${\tilde{S}}_{FU:k}^{\left(s\right)}(t)=P({\tilde{T}}_{FU:k,i}^{\left(s\right)}>t)$
	${\tilde{F}}_{FU:k}^{\left(s\right)}(t)=1-{\tilde{S}}_{FU:k}^{\left(s\right)}(t)$
	$S_{AE:k}(t)=P(T_{AE:k,i}>t)$
	$F_{AE:k}(t)=1-S_{AE:k}(t)$
Observation	${\hat{S}}_{k}^{\left(s\right)}(t)$ : Kaplan–Meier estimate for $S_{k}^{\left(s\right)}(t)$
	$n_{k}^{\left(s\right)}$ : the number of subjects
	$X_{k}^{\left(s\right)}$ : the number of AEs
	$a_{k}^{\left(s\right)}$ : the number of subjects at-risk at time *t*

Recall that we focus on literature-based meta-analysis, in which only information extracted from published papers is available. Thus, individual participant data are not available. We summarize data, which are supposed to be available in conducting the proposed method. Let $X_{k}^{\left(s\right)}$ be the number of subjects in the *k*th groups of the *s*th study, who experienced AEs during the study. We suppose $n_{k}^{\left(s\right)}$ and $X_{k}^{\left(s\right)}$ are available for all the studies. In addition, we assume that all the studies report the Kaplan–Meier estimate ${\hat{S}}_{k}^{\left(s\right)}(t)$ of the two treatment groups graphically, whose values are extracted from the graphical plot using some graph scan software such as the Digitizelt software (https://www.digitizeit.xyz/ja/). With them, we suppose that the survival function of the time to the end of the follow-up for AEs, $S_{FU:k}^{\left(s\right)}(t)$ are well estimated. Specifically, for the OS, $T_{FU:k,i}^{\left(s\right)}=T_{k,i}^{\left(s\right)}$ and then ${\hat{S}}_{k}^{\left(s\right)}(t)$ is used. For PFS, some additional follow-up duration $\tau^{\left(s\right)}$ (say, one month) is usually defined in the protocol and then $S_{FU:k}^{\left(s\right)}(t)$ is estimated by ${\hat{S}}_{k}^{\left(s\right)}(t-\tau^{\left(s\right)})$ . The resulting estimator for $S_{FU:k}^{\left(s\right)}(t)$ is denoted by ${\hat{S}}_{FU:k}^{\left(s\right)}(t)$ ; ${\hat{S}}_{FU:k}^{\left(s\right)}(t)={\hat{S}}_{k}^{\left(s\right)}(t)$ for the OS and ${\hat{S}}_{FU:k}^{\left(s\right)}(t)={\hat{S}}_{k}^{\left(s\right)}(t-\tau^{\left(s\right)})$ for the PFS. Let ${\hat{F}}_{FU:k}^{\left(s\right)}(t)=1-{\hat{S}}_{FU:k}^{\left(s\right)}(t)$ . To estimate $G_{k}^{\left(s\right)}(t)$ , the survival function of $C_{k,i}^{\left(s\right)}$ , we suppose that for each study, the number of subjects at risk for the efficacy endpoint is given for some time points. Let $a_{k}^{\left(s\right)}(t)$ be the number of subjects at-risk at *t*. Typically, it is given below the graphical plot of the Kaplan–Meier estimates for the efficacy endpoint. It is assumed that for the *k*th group of the *s*th study, $a_{k}^{\left(s\right)}(t)$ are available at $t=t_{1}^{\left(s\right)},t_{2}^{\left(s\right)},\ldots ,t_{K^{\left(s\right)}}^{\left(s\right)}$ , where $K^{\left(s\right)}$ is the number of time points for the *s*th study. For studies without $a_{k}^{\left(s\right)}(t)$ available, we suppose that the median follow-up time $m^{\left(s\right)}$ is available, which is defined as the sample median of ${\tilde{T}}_{k,i}^{\left(s\right)}=min(T_{k,i}^{\left(s\right)},C_{k,i}^{\left(s\right)})$ over all the subjects of the two groups in each individual study and sometimes is reported in literature to describe the status of the follow-up of the time-to-event endpoint as presented in Table 1.

## 3 Proposed methods

### 3.1 Methods under the independent follow-up duration

The idea of our proposed method is to summarize the occurrence of AEs by estimating the cumulative distribution function $F_{AE:k}(t)$ with information of the follow-up duration from the Kaplan–Meier estimates of the efficacy outcomes as the end of the follow-up of AEs. We begin with introducing our proposed method under a simpler setting of the independent follow-up duration. We assume that ${\tilde{T}}_{FU:k,i}^{\left(s\right)}$ and $T_{AE:k,i}^{\left(s\right)}$ are independent. Recall that ${\tilde{T}}_{FU:k,i}^{\left(s\right)}=min(T_{FU:k,i}^{\left(s\right)},C_{k,i}^{\left(s\right)})$ . The event that an AE is observed is equivalent to $T_{AE:k,i}^{\left(s\right)}$ occurring before the end of follow-up, ${\tilde{T}}_{FU:k,i}^{\left(s\right)}$ . Thus, the incidence proportion in each individual study is represented as $p_{k}^{\left(s\right)}=P\left(T_{AE:k,i}^{\left(s\right)}\leq {\tilde{T}}_{FU:k,i}^{\left(s\right)}\right)$ . By simple algebraic manipulation, with the assumption ${\tilde{T}}_{FU:k,i}^{\left(s\right)}⊥T_{AE:k,i}^{\left(s\right)}$ , it holds that

(1) $$\begin{array}{rl} p_{k}^{\left(s\right)} & =\int_{0}^{\infty }P\left(T_{AE:k,i}^{\left(s\right)}\leq t|{\tilde{T}}_{FU:k,i}^{\left(s\right)}=t\right)d{\tilde{F}}_{FU:k}^{\left(s\right)}(t)\\ & =\int_{0}^{\infty }P\left(T_{AE:k,i}^{\left(s\right)}\leq t\right)d{\tilde{F}}_{FU:k}^{\left(s\right)}(t)\\ & =\int_{0}^{\infty }F_{AE:k}(t)d\left(1-S_{FU:k}^{\left(s\right)}(t)G_{k}^{\left(s\right)}(t)\right). \end{array}$$

The cumulative distribution function $F_{AE:k}(t)$ is a key quantity to summarize the occurrence of AEs. Recall that we assume that it is common over the studies. We suppose some parametric model for $F_{AE:k}(t)$ , such as the exponential, the Weibull, and the log-normal distributions. The cumulative distribution function of the model is denoted by $F_{AE:k}(t;\theta_{k})$ , where $\theta_{k}$ is the unknown parameters. We estimate the censoring distribution $G_{k}^{\left(s\right)}(t)$ by assuming some parametric model $G_{k}^{\left(s\right)}(t;\eta_{k})$ . The unknown parameter $\eta_{k}$ is estimated by the method given in Section 3.2, and the resulting estimator is denoted by ${\hat{\eta }}_{k}$ . By replacing $S_{FU:k}^{\left(s\right)}(t)$ and $G_{k}^{\left(s\right)}(t)$ in (1) with ${\hat{S}}_{FU:k}^{\left(s\right)}(t)$ and $G_{k}^{\left(s\right)}\left(t;{\hat{\eta }}_{k}\right)$ , respectively, an empirical version of $p_{k}^{\left(s\right)}$ is given by

(2) $$\begin{array}{rl} p_{k}^{\left(s\right)}(\theta_{k}) & =\int_{0}^{\infty }F_{AE:k}(t;\theta_{k})d\left(1-{\hat{S}}_{FU:k}^{\left(s\right)}(t)G_{k}^{\left(s\right)}\left(t;{\hat{\eta }}_{k}\right)\right). \end{array}$$

Here, $\left(1-{\hat{S}}_{FU:k}^{\left(s\right)}(t)G_{k}^{\left(s\right)}\left(t;{\hat{\eta }}_{k}\right)\right)$ is a cumulative distribution function of a discrete distribution with masses at the jump points of ${\hat{S}}_{FU:k}^{\left(s\right)}(t)$ and the integral on the right-hand side of (2) is defined by the Riemann (Lebesgue)–Stieltjes integral. To be more specific, let the number of distinct time points, at which failure times of some subjects were observed, be denoted by $H^{\left(s\right)}$ , and the ordered observed failure times of the *s*th study be denoted by $0=w_{0}^{\left(s\right)}\leq w_{1}^{\left(s\right)}\leq \ldots w_{H^{\left(s\right)}}^{\left(s\right)}$ . Then, equation (2) is calculated by

$$\begin{array}{rl} p_{k}^{\left(s\right)}(\theta_{k}) & =\sum_{k=1}^{H^{\left(s\right)}}F_{AE:k}(w_{k-1}^{\left(s\right)};\theta_{k})\{{\hat{S}}_{FU:k}^{\left(s\right)}(w_{k-1}^{\left(s\right)})G_{k}^{\left(s\right)}\left(w_{k-1}^{\left(s\right)};{\hat{\eta }}_{k}\right)-{\hat{S}}_{FU:k}^{\left(s\right)}(w_{k}^{\left(s\right)})G_{k}^{\left(s\right)}\left(x_{k}^{\left(s\right)};{\hat{\eta }}_{k}\right)\}. \end{array}$$

Assuming $X_{k}^{\left(s\right)}∼$ Binomial( $n_{k}^{\left(s\right)}$ , $p_{k}^{\left(s\right)}(\theta_{k})$ ), we propose to estimate the unknown parameter $\theta_{k}$ by applying the maximum likelihood method. The likelihood function is defined by

∏s=1S(nk(s)Xk(s)){pk(s)(θk)}Xk(s){1−pk(s)(θk)}nk(s)−Xk(s).

Using the maximum likelihood estimator $\hat{\theta_{k}}$ , the cumulative distribution function of the occurrence of the AE before time *t* is estimated by $F_{AE:k}(t;{\hat{\theta }}_{k})$ and its 95% CIs is constructed as

$$\begin{array}{r} F_{AE:k}(t;{\hat{\theta }}_{k})\pm 1.96\times \frac{\sqrt{Var\left(F_{AE:k}(t;{\hat{\theta }}_{k})\right)}}{\sqrt{S}}, \end{array}$$

where the variance formula $Var\left(F_{AE:k}(t;\theta_{k})\right)$ is derived by using the delta method with the variance estimates of the maximum likelihood estimator.

### 3.2 Estimation of the censoring distribution

Recall that $a_{k}^{\left(s\right)}(t)$ is the number of subjects at-risk for the efficacy endpoint at *t*, which is available at $t=t_{1}^{\left(s\right)},t_{2}^{\left(s\right)},\ldots ,t_{K^{\left(s\right)}}^{\left(s\right)}$ . The proportion of subjects at-risk of the AE at time *t* is denoted by $q_{k}^{\left(s\right)}(t)=a_{k}^{\left(s\right)}(t)/n_{k}^{\left(s\right)}$ . We assume that $C_{k,i}^{\left(s\right)}$ follows the exponential distribution with the parameter $\eta_{k}$ and its survival function is denoted by $G_{k}^{\left(s\right)}(t;\eta_{k})$ . Under the assumption of independence between $T_{k,i}^{\left(s\right)}$ and $C_{k,i}^{\left(s\right)}$ , $q_{k}^{\left(s\right)}(t)=S_{k}^{\left(s\right)}(t)G_{k}^{\left(s\right)}\left(t;\eta_{k}\right)$ holds. It motivates us to estimate the unknown parameter $\eta_{k}$ by applying the least square method for each study separately:

$$\begin{array}{r} minimize\sum {\left\{q_{k}^{\left(s\right)}(t)-{\hat{S}}_{k}^{\left(s\right)}(t)G_{k}^{\left(s\right)}\left(t;\eta_{k}\right)\right\}}^{2}, \end{array}$$

where the summation is taken over $t=t_{1}^{\left(s\right)},t_{2}^{\left(s\right)},\ldots ,t_{K^{\left(s\right)}}^{\left(s\right)}$ .

For studies without $a_{k}^{\left(s\right)}(t)$ reported, the following approach can be taken. Suppose the median follow-up time $m^{\left(s\right)}$ is reported for the primary endpoint, which is defined by a simple sample median of ${\tilde{T}}_{k,i}^{\left(s\right)}=min(T_{k,i}^{\left(s\right)},C_{k,i}^{\left(s\right)})$ over the subjects of the two treatment groups for the *s*th study. It is often done as presented in Table 1. Note that the median follow-up time is reported with various definitions.^12^ Suppose the censoring distribution is common over the two treatment groups, and the survival function of the censoring is denoted by $G^{\left(s\right)}(t)$ . We suppose a parametric model $G^{\left(s\right)}(t;\eta )$ for G(t) . Since we assume that the independence between $T_{k,i}^{\left(s\right)}$ and $C_{k,i}^{\left(s\right)}$ , $P({\tilde{T}}_{k,i}^{\left(s\right)}>t)=S^{\left(s\right)}(t)G^{\left(s\right)}(t;\eta )$ holds, where $S^{\left(s\right)}(t)$ is the marginal survival function of the *s*th study and can be estimated by ${\hat{S}}^{\left(s\right)}(t)=\frac{n_{0}^{\left(s\right)}}{n_{1}^{\left(s\right)}+n_{0}^{\left(s\right)}}{\hat{S}}_{0}^{\left(s\right)}(t)+\frac{n_{1}^{\left(s\right)}}{n_{1}^{\left(s\right)}+n_{0}^{\left(s\right)}}{\hat{S}}_{1}^{\left(s\right)}(t)$ . Then, since we assume that $C_{k,i}^{\left(s\right)}$ follows the exponential distribution with the hazard parameter η , we can determine η by solving $0.5={\hat{S}}^{\left(s\right)}(m^{\left(s\right)})exp(-\eta m^{\left(s\right)})$ .

In the above argument, we assumed that $C_{k,i}^{\left(s\right)}$ follows the exponential distribution, which had a single parameter. For the studies with $a_{k}^{\left(s\right)}(t)$ observed at multiple time points, distributions with multi-dimensional parameters can be handled in a similar way.

### 3.3 Methods under the dependent follow-up duration

The independent follow-up assumption ${\tilde{T}}_{FU:k,i}^{\left(s\right)}⊥T_{AE:k,i}^{\left(s\right)}$ in Section 3.1 may not hold in practice. This section considers an approach not requiring the independence assumption. By simple algebraic manipulation, the incidence proportion ${\tilde{p}}_{k}^{\left(s\right)}=P\left(T_{AE:k,i}^{\left(s\right)}\leq {\tilde{T}}_{FU:k,i}^{\left(s\right)}\right)$ is represented as

(3) $$\begin{array}{r} {\tilde{p}}_{k}^{\left(s\right)}=\int_{0}^{\infty }\frac{P\left(T_{AE:k,i}^{\left(s\right)}\leq t,{\tilde{T}}_{FU:k,i}^{\left(s\right)}=t\right)}{P\left({\tilde{T}}_{FU:k,i}^{\left(s\right)}=t\right)}d\left(1-S_{FU:k}^{\left(s\right)}(t)G_{k}^{\left(s\right)}(t)\right). \end{array}$$

We propose to model the joint probability $P\left(T_{AE:k,i}^{\left(s\right)}\leq t_{1},{\tilde{T}}_{FU:k,i}^{\left(s\right)}\leq t_{2}\right)$ in (3) using a two-dimensional parametric copula, such as Frank copula,^13^ Clayton–Cook–Johnson copula,^14^ ^,^ ^15^ and Gumbel–Hougaard copula.^16^ Recall that $F_{AE:k}\left(t\right)=P\left(T_{AE:k,i}^{\left(s\right)}\leq t\right)$ and denote ${\tilde{F}}_{FU:k}^{\left(s\right)}(t)=P\left({\tilde{T}}_{FU:k,i}^{\left(s\right)}\leq t\right)$ . Thus, we model the joint cumulative distribution function by $P\left(T_{AE:k,i}^{\left(s\right)}\leq t_{1},{\tilde{T}}_{FU:k,i}^{\left(s\right)}\leq t_{2}\right)=C_{k,\psi_{k}}(F_{AE:k}(t_{1}),{\tilde{F}}_{FU:k}^{\left(s\right)}(t_{2}))$ , where $C_{k,\psi_{k}}(u,v)$ is a parametric copula model for the *k*th group, $\psi_{k}$ is an unknown parameter in the copula; $C_{k,\psi_{k}}(u,v)=\psi_{k}^{-1}log\{1+\frac{\left(\exp(-\psi_{k}u)-1)(\exp(-\psi_{k}v)-1\right)}{\exp(-\psi_{k}u)+1}\}$ with $\psi_{k}\neq 0$ for Frank copula, $C_{\psi_{k}}(u,v)=(u^{-\psi_{k}}+v^{-\psi_{k}})$ with $-1\leq \psi_{k},\psi_{k}\neq 0$ for Clayton–Cook–Johnson copula, and $C_{k,\psi_{k}}(u,v)=exp\{-[(-log(u){)}^{\psi }+(-log(v){)}^{\psi_{k}}{]}^{1/\psi_{k}}\}$ with $-1\leq \psi_{k}$ for Gumbel–Hougaard copula. By simple algebra, it holds that

$$\begin{array}{r} P\left(T_{AE:k,i}^{\left(s\right)}\leq t_{1},{\tilde{T}}_{FU:k,i}^{\left(s\right)}=t_{2}\right)=\frac{d}{dv}C_{k,\psi_{k}}(F_{AE:k}(t_{1}),v){|}_{v={\tilde{F}}_{FU:k}^{\left(s\right)}(t_{2})}\times \frac{d}{dt_{2}}{\tilde{F}}_{FU:k}^{\left(s\right)}(t_{2}). \end{array}$$

Then (3) is expressed as

(4) $$\begin{array}{r} {\tilde{p}}_{k}^{\left(s\right)}=\int_{0}^{\infty }\frac{\partial }{\partial u}C_{k,\psi_{k}}\left(F_{AE:k}(t),v\right){|}_{v=\left(1-S_{FU:k}^{\left(s\right)}(t)G_{k}^{\left(s\right)}(t)\right)}d\left(1-S_{FU:k}^{\left(s\right)}(t)G_{k}^{\left(s\right)}(t)\right). \end{array}$$

As done in the case of the independent follow-up, by replacing $S_{k}^{\left(s\right)}(t)$ and $G_{k}^{\left(s\right)}(t)$ in (4) with ${\hat{S}}_{k}^{\left(s\right)}(t)$ and $G_{k}^{\left(s\right)}(t;{\hat{\eta }}_{k})$ , respectively, an empirical version of ${\tilde{p}}_{k}^{\left(s\right)}$ is given by

$$\begin{array}{r} {\tilde{p}}_{k}^{\left(s\right)}(\theta_{k},\psi_{k})=\int_{0}^{\infty }\frac{\partial }{\partial u}C_{k,\psi_{k}}\left(F_{AE:k}(t;\theta_{k}),v\right){|}_{v=(1-{\hat{S}}_{FU:k}^{\left(s\right)}(t)G_{k}^{\left(s\right)}(t;{\hat{\eta }}_{k}))}d\left(1-{\hat{S}}_{k}^{\left(s\right)}(t)G_{k}^{\left(s\right)}(t;{\hat{\eta }}_{k})\right). \end{array}$$

Assuming $X_{k}^{\left(s\right)}∼$ Binomial( $n_{k}^{\left(s\right)}$ , ${\tilde{p}}_{k}^{\left(s\right)}(\theta_{k},\psi_{k})$ ), one can estimate unknown parameters $\theta_{k}$ and $\psi_{k}$ and conduct statistical inference following the maximum likelihood method.

To obtain the maximum likelihood estimators in Sections 3.1 and 3.3, since ${\tilde{p}}_{k}^{\left(s\right)}(\theta_{k})$ and ${\tilde{p}}_{k}^{\left(s\right)}(\theta_{k},\psi_{k})$ are a non-linear function with respect to unknown parameters, we need to apply non-linear optimization. We used *NLMIXED* procedure in *SAS* (SAS Institute Co. Ltd.). An SAS code to implement the proposed method is available at 10.5281/zenodo.16872253.

## 4 Example: Re-analysis of the motivating meta-analysis

We illustrate our proposed method by re-analyzing the motivating meta-analysis dataset in Section 2.1. As presented in Table 1, among the 17 studies in the meta-analysis, 12 studies had the PFS or OS as the primary endpoint and reported the Kaplan–Meier estimates of its survival function for the two treatment groups as a graphical display. Among them, the study 11 reported neither the number of at-risk nor the median follow-up time for the primary endpoint. We exclude the study 11 in our illustration. The studies 3 and 5 were designed as a study with the two bevacizumab groups and a control one. In this illustration, we included the two bevacizumab groups of these studies separately in the model and regarded them as independent studies for illustration. Then, there were 13 studies for the both groups. For these studies, to determine the censoring distribution, we suppose the exponential distribution for $C_{k,i}^{\left(s\right)}$ . The unknown parameter $\eta_{k}$ was estimated by the least-square method for the number of subjects at-risk in Section 3.2 if it was available. For the studies without the number of subjects at-risk, we utilized the median follow-up time to this purpose by the method in Section 3.2.

**Figure 1:**
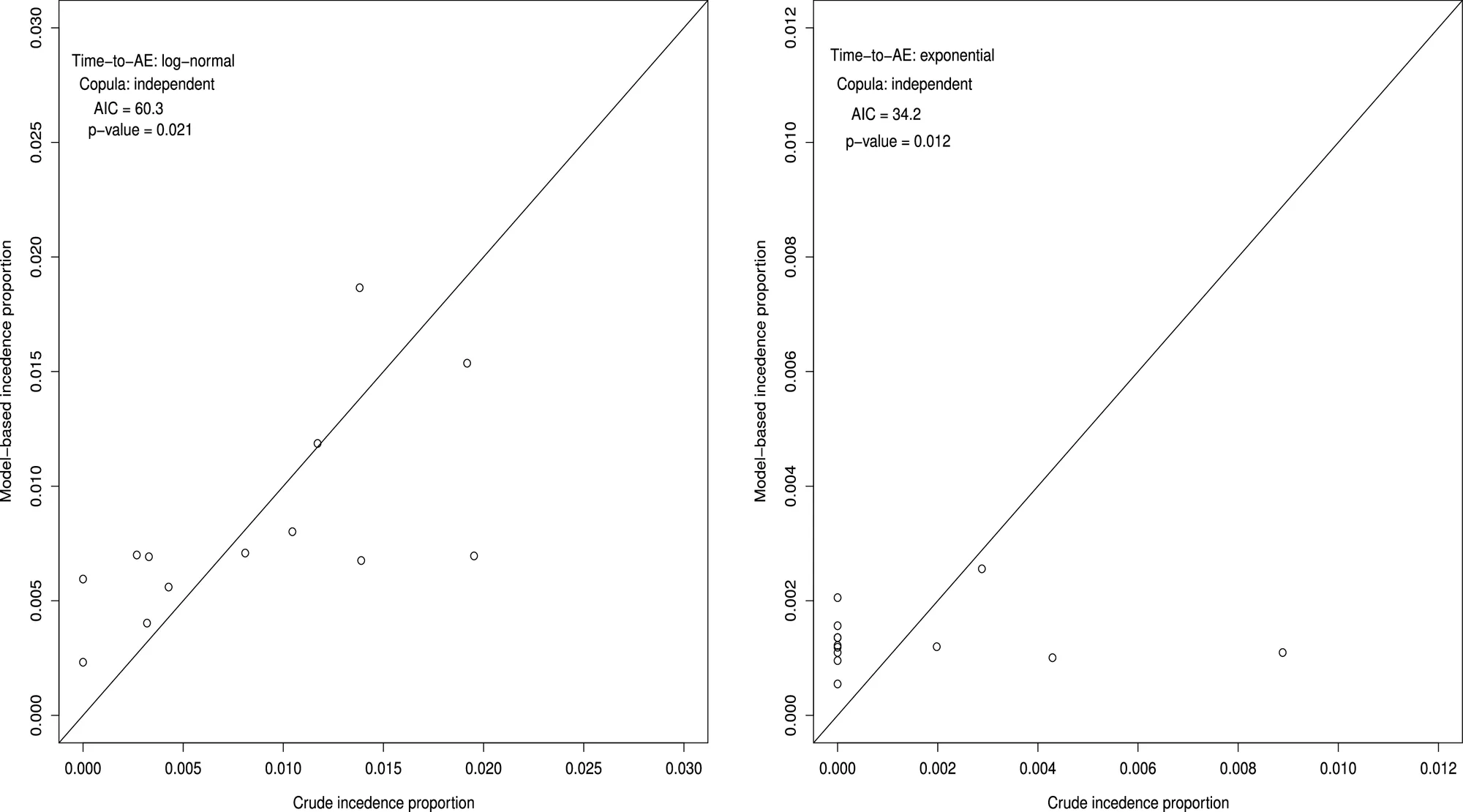
Goodness-of-fit of the best model under the independent follow-up duration for the bevacizumab group (S = 13, left panel) and the control group (S = 13, right panel); p-value is for the Pearson chi-squared goodness-of-fit test. Figure 1 Long description.

At first, we apply the proposed method under the independence follow-up assumption. We considered three candidate models for $T_{AE:k,i}^{\left(s\right)}$ ; we assumed it followed the exponential, the Weibull, or the log-normal distributions. To select the most relevant model, we calculated the Akaike information criterion (AIC) for each model; AIC =-2 loglikelihood +2*p*, where *p* is the number of unknown parameters to estimate.^17^ The model with smaller AIC is regarded as fitting better to data. The AICs of the candidate distributions are shown in Table 3. For the bevacizumab group, the log-normal distribution had the smallest AIC, suggesting that the bevacizumab group exhibited a better fit when assuming it, while the control group an exponential distribution. To further evaluate the goodness-of-fit of the selected model, we calculated and contrasted the model-based incidence proportion with the crude one. The model-based incidence proportion is defined as

$$\begin{array}{r} p_{k}^{(s)*}=\int_{0}^{\infty }F_{AE:k}(t;{\hat{\theta }}_{k})d\left(1-{\hat{S}}_{FU:k}^{\left(s\right)}(t)G_{k}^{\left(s\right)}\left(t;{\hat{\eta }}_{k}\right)\right) \end{array}$$

and the crude one as ${\hat{p}}_{k}^{\left(s\right)}=X_{k}^{\left(s\right)}/n_{k}^{\left(s\right)}$ . In Figure 1, pairs of ( ${\hat{p}}_{k}^{\left(s\right)}$ , $p_{k}^{(s)*}$ ) are plotted, for the bevacizumab (left panel) and the control (right panel) groups, respectively. The plots should lie around the diagonal line when the model successfully captures the true structure. To make a formal evaluation of the goodness-of-fit, one may use chi-squared goodness-of-fit test. Let

(5) $$\begin{array}{r} Z_{GOF,k}^{2}=\sum_{s=1}^{S}\frac{(X_{k}^{\left(s\right)}-n_{k}^{\left(s\right)}p_{k}^{(s)*}{)}^{2}}{n_{k}^{\left(s\right)}p_{k}^{(s)*}}. \end{array}$$

Note that $n_{k}^{\left(s\right)}p_{k}^{(s)*}$ is the expected number of the occurrence of the AE if the fitted model is correctly specified. Under the null hypothesis that the model is correctly specified, $Z_{GOF,k}^{2}$ follows the chi-squared distribution of the degree of freedom S−1 . It attaches a quantitative measure of the goodness-of-fit. Figure 1 suggests that the bevacizumab group generally showed a good fit, while the control group exhibited slightly poorer. On the other hand, the *p*-value of the goodness-of-fit test for the bevacizumab and the control groups were *p* = 0.021 and *p* = 0.012, respectively. Thus, the goodness-of-fit was not satisfactory in the both groups. The observed poor fit in the control group can be attributed to the fact that there were several trials in the control group (9 out of 13 trials) with no AEs reported.

**Table 3:** Akaike information criterion (AIC) for comparison of models for the distribution of the time-to-AE and the copula for dependent follow-up in the meta-analysis of the motivating data; NA, not available due to non-convergence of non-linear optimization to maximize the log-likelihood function Table 3 Long description.

		Bevacizumab group			Control group		
Follow-up	Copula	Exponential	Weibull	Log-normal	Exponential	Weibull	Log-normal
Independent	Independent	62.3	60.7	60.7	34.2	NA	NA
							
Dependent	Clayton	63.4	63.4	63.5	36.0	NA	35.6
	Frank	64.0	62.4	62.1	35.1	NA	NA
	Gumbel	64.4	62.1	61.6	33.9	35.9	45.7

**Figure 2:**
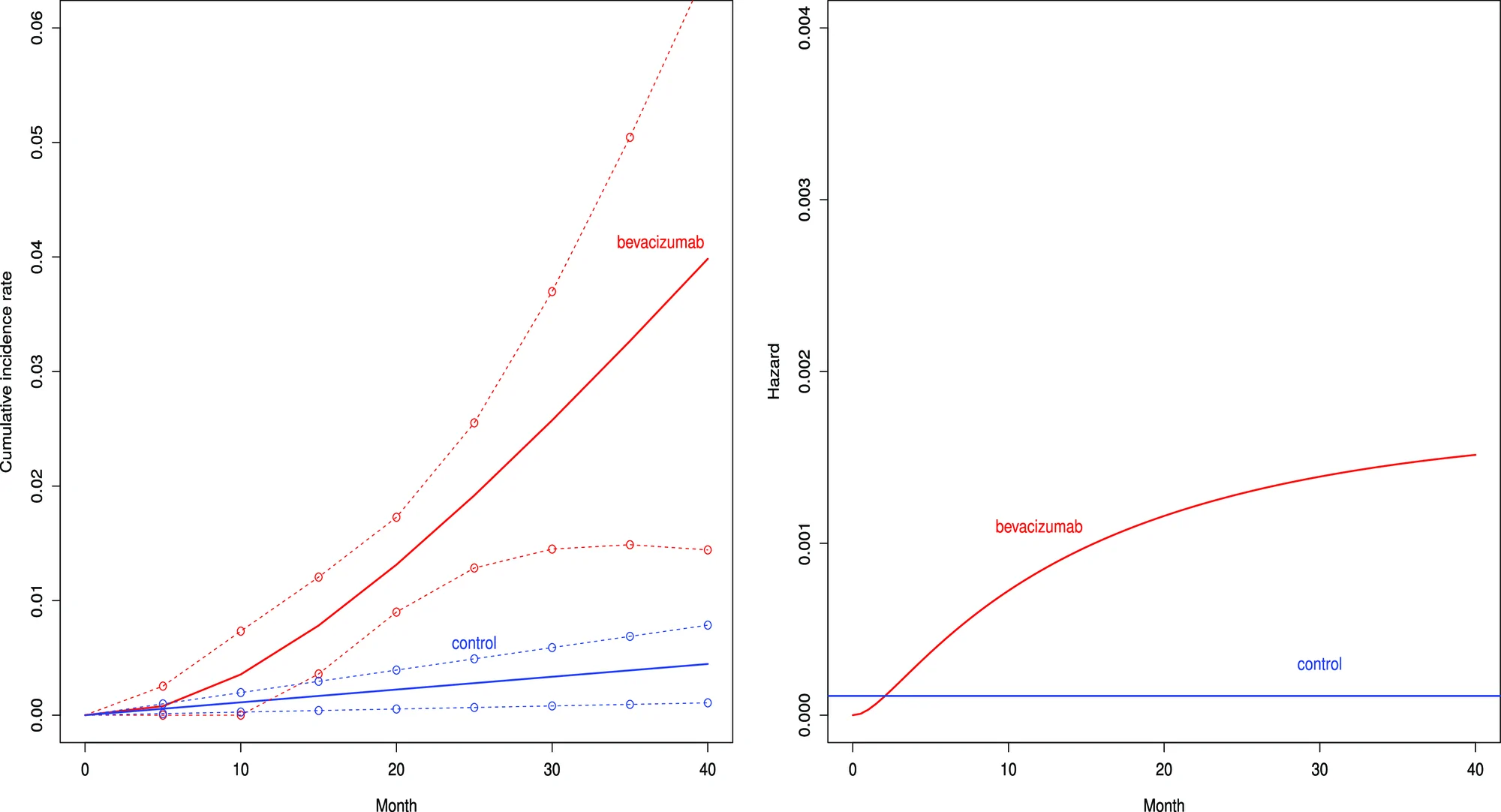
Estimated cumulative incidence functions $F_{AE:k}(t;{\hat{\theta }}_{k})$ (solid curve) with point-wise two-sided 95 % confidence bands (broken curve) of the bevacizumab and the control groups (left panel) and the corresponding hazard functions (right panel) under the independent follow-up duration. Figure 2 Long description.

Figure 2 depicts the cumulative incidence proportion of AEs, $F_{AE:k}(t)$ , (left panel) and the corresponding hazard function (right panel) when fitting the distribution with the best AIC for both groups. The cumulative incidence proportion was higher uniformly over time in the bevacizumab groups. The exponential distribution was selected for the control group, and then the hazard of the occurrence of the AE was constant over time. On the other hand, the hazard of AEs increased in the bevacizumab group (see the right panel of Figure 2).

**Figure 3:**
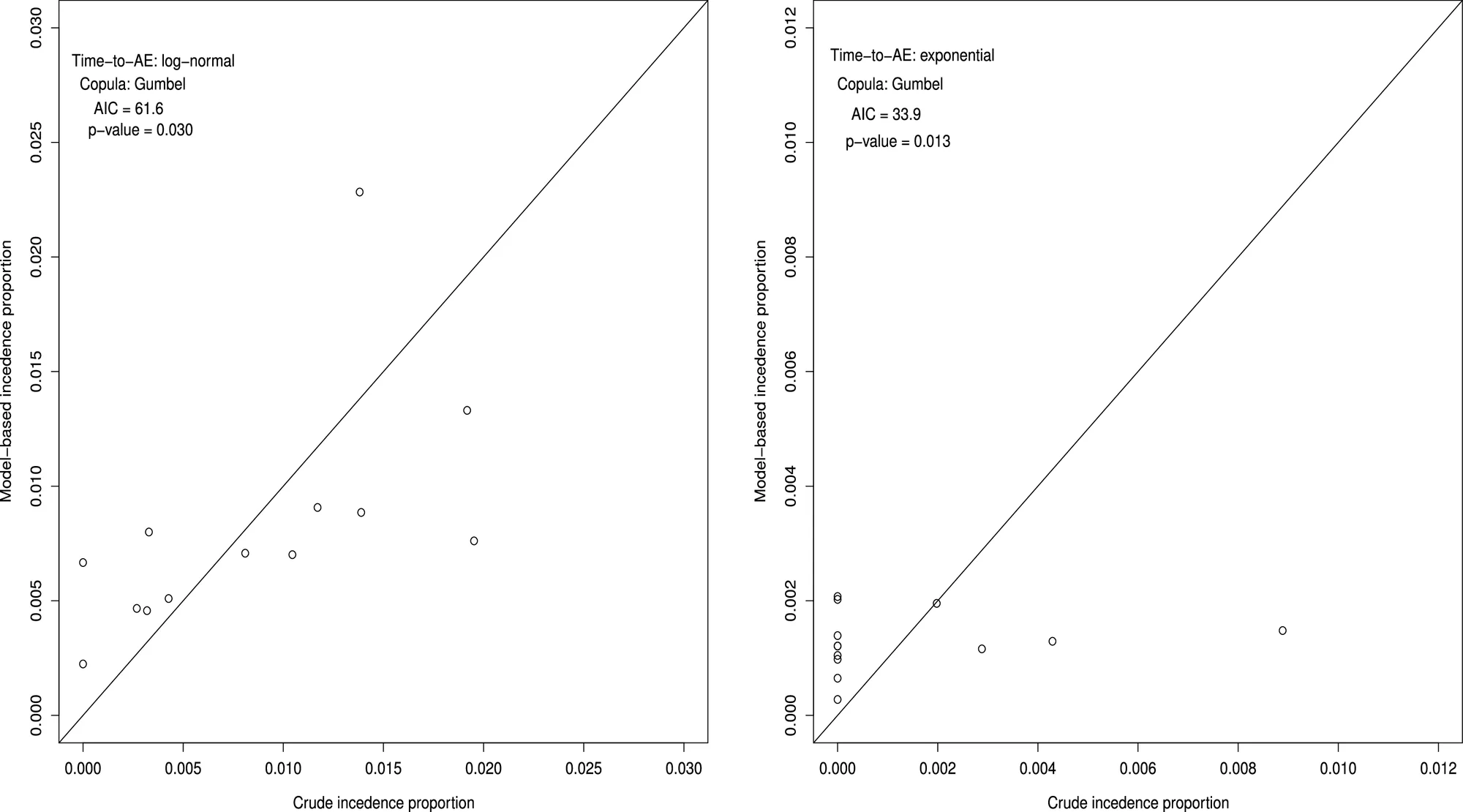
Goodness-of-fit of the best model under the dependent follow-up duration for the bevacizumab group (S = 13, left panel) and the control group (S = 13, right panel); p-value is for the Pearson chi-squared goodness-of-fit test. Figure 3 Long description.

**Figure 4:**
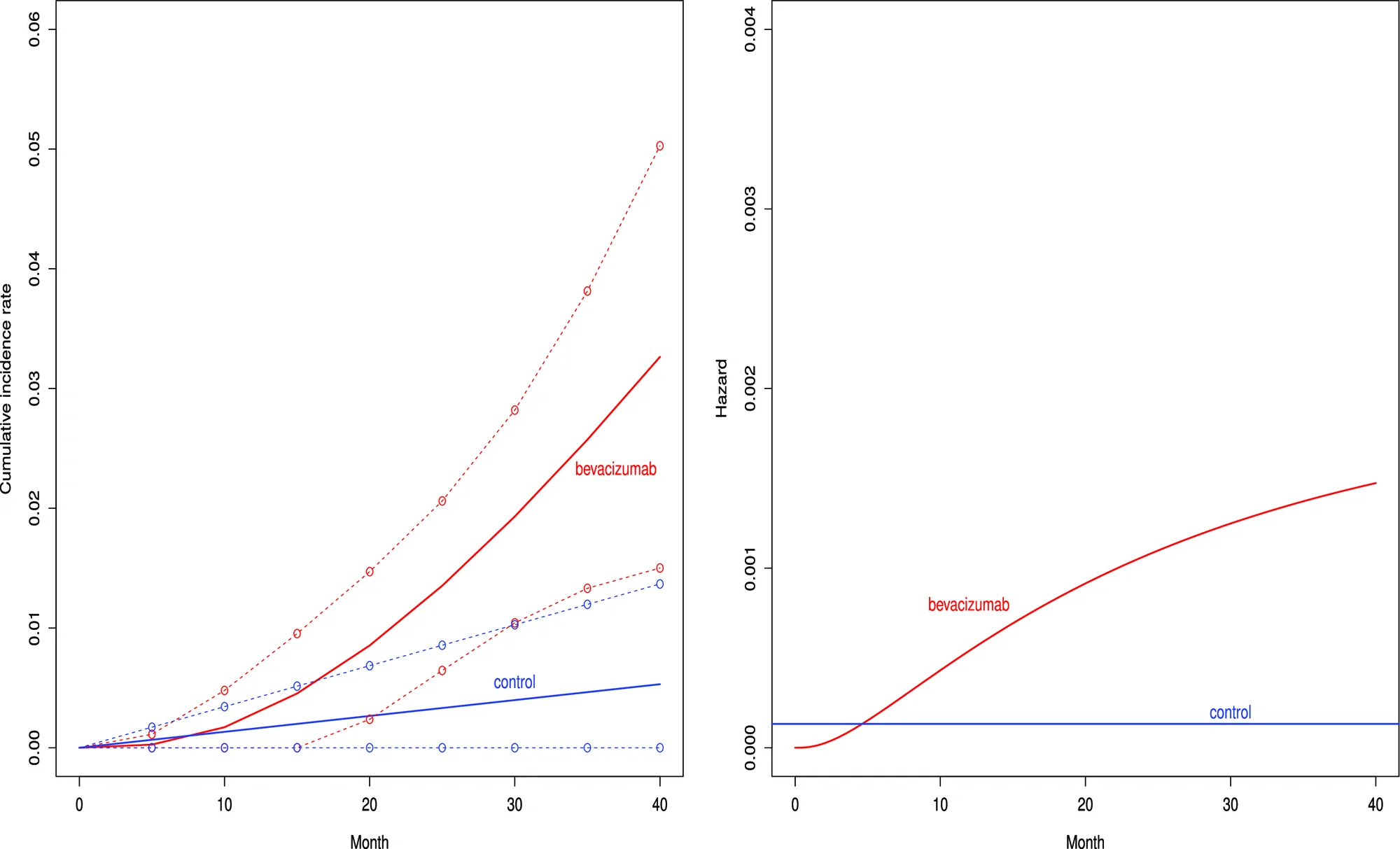
Estimated cumulative incidence functions $\;F_{AE:k}(t;{\hat{\theta }}_{k})$ (solid curve) with point-wise two-sided 95 % confidence bands (broken curve) of the bevacizumab and the control groups (left panel) and the corresponding hazard functions (right panel) under the dependent follow-up duration. Figure 4 Long description.

Next, we conducted analyses under the dependent follow-up duration. For the copula function $C_{k,\psi_{k}}(u,v)$ , we consider Frank copula, Clayton–Cook–Jonhson copula, and Gumbel–Hougaard copula as candidate models. In Table 3, we report the AICs for the candidate models. For the bevacizumab group, the best fit was observed when assuming the log-normal distribution for $F_{AE:k}(t)$ and the Gumbel–Hougaard family for the copula function. For the control group, the best fit was found when assuming the exponential distribution and the Gumbel–Hougaard family. As done under the independent follow-up case, the goodness-of-fit was assessed by plotting the model-based incidence proportion and the crude one. The model-based incidence proportion was defined as

$$\begin{array}{r} {\tilde{p}}_{k}^{(s)*}=\int_{0}^{\infty }\frac{\partial }{\partial v}C_{k,{\hat{\psi }}_{k}}\left(F_{AE:k}(t;{\hat{\theta }}_{k}),v\right){|}_{v=\left(1-{\hat{S}}_{k}^{\left(s\right)}(t)G_{k}^{\left(s\right)}\left(t;{\hat{\eta }}_{k}\right)\right)}d\left(1-{\hat{S}}_{FU:k}^{\left(s\right)}(t)G_{k}^{\left(s\right)}\left(t;{\hat{\eta }}_{k}\right)\right), \end{array}$$

and the plot is given in Figure 3 for the bevacizumab group (left panel) and the control group (right panel), respectively. A similar goodness-of-fit test can be constructed by replacing $p_{k}^{(s)*}$ with ${\tilde{p}}_{k}^{(s)*}$ in (5). For the bevacizumab group, the best model under the dependent follow-up duration had a slightly larger AIC value than the best model under the independent follow-up duration. Correspondingly, Figure 3 suggested a similar goodness-of-fit with a significance *p*-value of 0.030 for the Pearson chi-squared goodness-of-fit test. For the control group, AIC was also not improved accounting for dependence, and Figure 3 suggested unsatisfactory fit to the data with the *p*-value 0.013 of the goodness-of-fit test. Although these observations necessitate further improvement of the models, we compare the occurrence of AEs between the two treatment groups using the best-performing models. In Figure 4, we present the cumulative incidence functions of the two groups (left panel) and the corresponding hazard functions (right panel). The confidence interval of the cumulative incidence function was much wider under the dependent follow-up duration. In the control group, some studies did not report any occurrence of the AE. Thus, the model under dependent follow-up duration had extra parameters in the copula model and then estimation was instable with limited information. In Table 4, we present the RR of the bevacizumab group to the control with respect to the cumulative incidence up to selected time points with the best models of each group. As suggested by Figures 2 and 4, the hazard of the AE in the bevacizumab increased over time. Correspondingly, significance differences of the cumulative incidence were observed consistently after *t* = 30. Recall that the aggregated RR by the Mantel–Haenszel method was 3.28 (95 % CI: 1.97–5.48). Table 4 indicates that the RR was not homogeneous over time.

**Table 4:** The relative risk of the cumulative occurrence of the AE until selected time points by the model minimizing the AIC under the independent and the dependent follow-up durations Table 4 Long description.

	Independent		Dependent	
*t*	RR (95 % CI)	*P*-value	RR (95 % CI)	*P*-value
5	1.404(0.134,14.738)	0.777	0.394(0.010,15.183)	0.617
10	3.178(0.860,11.739)	0.083	1.289(0.118,14.089)	0.835
15	4.664(1.835,11.854)	0.001	2.273(0.331,15.633)	0.404
20	5.875(2.581,13.377)	< 0.001	3.215(0.566,18.271)	0.188
25	6.865(2.998,15.720)	< 0.001	4.077(0.772,21.530)	0.098
30	7.679(3.197,18.443)	< 0.001	4.851(0.936,25.128)	0.060
35	8.354(3.282,21.268)	< 0.001	5.540(1.063,28.870)	0.042
40	8.918(3.308,24.041)	< 0.001	6.153(1.160,32.619)	0.033

## 5 Simulation study

### 5.1 Data generation and applied methods

To evaluate the validity of the method proposed in Section 3, we conducted a simulation study. The setting for the simulation study was designed mimicking the dataset of Zhu et al.^1^ We only considered the bevacizumab group (only k=1 ) since our proposal was to fit the model separately by the group. We selected 13 studies with the median time of the primary time-to-event endpoint reported. The median survival times for the selected 13 studies are listed Table 5. In generating simulation dataset, although the median survival time was for the OS in some studies, we regarded it as the time for the PFS for simplicity and assume that the PFS was the primary endpoint and the follow-up of the AEs were made until the PFS. We suppose that the Kaplan–Meier estimate for the PFS was available and the at-risk data were available at three time points (6, 12, and 18 months) for all the studies. Two setting on the follow-up duration for AEs were considered: (1) the independent follow-up duration (Dataset 1) and (2) the dependent follow-up duration (Dataset 2). For each setting, we generated 1,000 meta-analyses of 13 studies, whose sample size of each group was the same as the bevacizumab group of each study in Table 1 (see Table 5).

**Table 5:** List of parameters of studies used in generating simulation datasets: *n* shows the number of subjects for efficacy analysis in the bevacizumab group; $\lambda^{\left(s\right)}$ : the hazard of the exponential distribution for PFS; and $\lambda_{C}^{\left(s\right)}$ : the hazard of the exponential distribution for censoring Table 5 Long description.

study	Authors	*n*	Median PFS	$\lambda^{\left(s\right)}$	$\lambda_{C}^{\left(s\right)}$
1	Aghajanian, 2012	242	12.4	0.0243	0.02
3-1	Burger, 2011	625	11.2	0.0269	0.015
3-2	Burger, 2011	623	14.1	0.0213	0.015
4	Chinot, 2014	458	10.6	0.0284	0.025
5-1	Giantonio, 2007	237	2.7	0.1115	0.01
5-2	Giantonio, 2007	280	7.3	0.0412	0.015
6	Herbst, 2011	319	3.4	0.0885	0.018
8	Kelly, 2012	524	9.9	0.0304	0.02
9	Kim, 2012	143	5.6	0.0538	0.012
11	Kindler, 2010	300	3.8	0.0792	0.01
12	Miller, 2007	347	11.8	0.0255	0.024
14	Perren, 2011	764	19.8	0.0152	0.015
17	Sandler, 2006	417	6.2	0.0486	0.01

*Dataset 1A: Independence*

We assumed that $T_{AE:k,i}^{\left(s\right)}$ followed an exponential distribution with a hazard of $\lambda_{AE}$ . Three settings of $\lambda_{AE}$ were considered; $\lambda_{AE}=0.002$ (scenario 1), =0.01 (scenario 2), and =0.05 (scenario 3). The distribution of $T_{k,i}^{\left(s\right)}$ was supposed as an exponential distribution, whose hazard $\lambda^{\left(s\right)}$ was set based on the median PFS in Table 5. The censoring for the PFS, $C_{k,i}^{\left(s\right)}$ was generated from an exponential distribution, whose hazard $\lambda_{C}^{\left(s\right)}$ was set a study-specific value listed in Table 5. Then, we set ${\tilde{T}}_{k,i}^{\left(s\right)}=min(T_{k,i}^{\left(s\right)},C_{k,i}^{\left(s\right)})$ and $\Delta_{k,i}^{\left(s\right)}=I(T_{k,i}^{\left(s\right)}\leq C_{k,i}^{\left(s\right)})$ and the occurrence of AE was determined by whether $T_{AE:k,i}^{\left(s\right)}\leq {\tilde{T}}_{k,i}^{\left(s\right)}$ met or not. With these individual-level simulated data, we calculate the Kaplan–Meier estimate for the PFS and the number of AEs observed within the follow-up duration, which is denoted as $X_{k}^{\left(s\right)}$ . The number of at-risk for the PFS, $a_{k}^{\left(s\right)}(t)$ was calculated at t=6,12,18 . We regard them as data.

*Dataset 2A: Dependence*

We assume the same three scenarios for $\lambda_{AE}$ as Dataset 1. For the marginal distribution of $T_{k,i}^{\left(s\right)}$ and $C_{k,i}^{\left(s\right)}$ , the same exponential parameters $\lambda^{\left(s\right)}$ and $\lambda_{C}^{\left(s\right)}$ were considered, respectively, as given in the case of the independent follow-up duration (see Table 5). From the independence assumption between $T_{k,i}^{\left(s\right)}$ and $C_{k,i}^{\left(s\right)}$ , the cumulative distribution function of ${\tilde{T}}_{FU:k,i}^{\left(s\right)}=min(T_{k,i}^{\left(s\right)},C_{k,i}^{\left(s\right)})$ is given by ${\tilde{F}}_{FU,k}^{\left(s\right)}=1-exp(-t/(\lambda^{\left(s\right)}+\lambda_{C}^{\left(s\right)}))$ . We assumed that the dependence between $T_{AE:k,i}^{\left(s\right)}$ and ${\tilde{T}}_{FU:k,i}^{\left(s\right)}$ was determined by the copula function. We considered three copulas: Clayton–Cook–Johnson, Gumbel–Hougaard, and Frank copulas. We set the parameter $\psi_{K}$ in each copula so that the resulting Kendall’s τ values were 0.2 or 0.5. We generated $T_{AE:k,i}^{\left(s\right)}$ and ${\tilde{T}}_{FU:k,i}^{\left(s\right)}$ from the joint distribution defined by the assumed copula with the supposed marginal distributions of $T_{AE:k,i}^{\left(s\right)}$ and ${\tilde{T}}_{FU:k,i}^{\left(s\right)}$ according to the method given in Tsukahara.^18^ We set ${\tilde{T}}_{k,i}^{\left(s\right)}={\tilde{T}}_{FU:k,i}^{\left(s\right)}$ . By setting $\Delta_{k,i}^{\left(s\right)}=1$ with the probability $P(T_{k,i}^{\left(s\right)}\leq C_{k,i}^{\left(s\right)})$ and otherwise $\Delta_{k,i}^{\left(s\right)}=0$ , we created the observation ( ${\tilde{T}}_{k,i}^{\left(s\right)}$ , $\Delta_{k,i}^{\left(s\right)}$ ) for the PFS. The occurrence of the AE was also determined according to $T_{AE:k,i}^{\left(s\right)}\leq {\tilde{T}}_{k,i}^{\left(s\right)}$ . Then, the marginal survival function for $T_{k,i}^{\left(s\right)}$ was estimated by the Kaplan–Meier method and the number of AEs $X_{k}^{\left(s\right)}$ was calculated. The number of at-risk for the PFS, $a_{k}^{\left(s\right)}(t)$ was calculated at t=6,12,18 .

We conducted an additional simulation study with *S* = 30. The number of subjects in each study was generated from the discrete uniform distribution on [50, 500]. The other settings were not altered. We generated datasets under the independent and dependent follow-up durations and the corresponding datasets were called Datasets 1B and 2B, respectively.

In the setting explained above, the at-risk data were available at 6, 12, and 18 months for all the studies. It is called Case 1. To evaluate impacts of availability of the at-risk data, in addition to Case 1, we consider an alternative setting, in which the at-risk data were available at two time points randomly selected on {2,3,…,15} for each study. It is called Case 2. We evaluate performance of the method in Section 3.2 in estimating the exponential parameter for the study-specific censoring distribution for Cases 1 2.

### 5.2 Results

We analyzed both Datasets 1A and 2A using the two approaches: the method in Section 3.1 (independent follow-up duration) and that in Section 3.3 (dependent follow-up duration). In both Datasets 1A and 2A, the marginal distribution for the time until the first occurrence of the AE $T_{AE:k,i}^{\left(s\right)}$ was the exponential distribution with the hazard $\lambda_{AE}$ . For each of the three scenarios of $\lambda_{AE}$ (scenario 1, $\lambda_{AE}=0.002$ ; scenario 2, $\lambda_{AE}=0.01$ ; and scenario 3; $\lambda_{AE}=0.05$ ), we fit the exponential distribution and evaluated bias and coverage probability (CP) on estimation of $\lambda_{AE}$ . Recall that the target estimand of our proposed method is the cumulative distribution function $F_{AE:k}(t)$ . Since $T_{AE:k,i}^{\left(s\right)}$ was generated from the exponential distribution, it is given by $F_{AE:k}(t)=1-exp(-\lambda_{AE}t)$ . Then, we evaluate estimates for $\lambda_{AE}$ . To see how likely the AE occurred in each scenario, the average of the observed incidence proportion was calculated over 1,000 simulated datasets for Dataset 1; they were 0.023, 0.116, and 0.556 for scenarios 1–3, respectively.

**Table 6:** Summary of estimates (median (q1, q3)) and empirical coverage probabilities (CP) of the exponential parameter for the time-to-AE and frequencies that each model was selected with AIC for Dataset 1A (*S* = 13, independence): True implies the value of $\lambda_{AE}(\times 100)$ , and NoC is the number of realizations with successful convergence in iteration for the maximum likelihood estimation Table 6 Long description.

	Data		Model			
Parameter	Scenario	True	Independent	Frank	Clayton	Gumbel
$100\times \lambda_{AE}$	1	0.2	0.199 (0.187, 0.212)	0.158 (0.068, 0.582)	0.224 (0.199, 0.302)	0.238 (0.203, 0.518)
	2	1	0.998 (0.968, 1.026)	1.006 (0.739, 1.267)	1.038 (0.993, 1.134)	1.060 (1.006, 1.317)
	3	5	4.980 (4.899, 5.062)	4.994 (4.843, 5.139)	5.041 (4.946, 5.153)	5.102 (5.014, 5.216)
						
CP (NoC)	1	0.95	0.960 (960)	0.735 (546)	0.976 (911)	0.974 (526)
	2	0.95	0.975 (975)	0.890 (884)	0.976 (959)	0.948 (494)
	3	0.95	0.983 (983)	0.987 (987)	0.977 (959)	0.969 (818)
						
Selected by AIC	1		790	144	39	27
	2		832	91	38	39
	3		857	68	26	49

**Table 7:** Summary of estimates (median (q1,q3)) and empirical coverage probabilities (CP) of the exponential parameter and frequencies that each model was selected with AIC for the time-to-AE for Dataset 2A (*S* = 13, dependence): TRUE implies the value of $\lambda_{AE}(\times 100)$ , dep. shows the applied copula, and NoC is the number of realizations with successful convergence in iteration for the maximum likelihood estimation Table 7 Long description.

Parameter	scn.	TRUE	dep.	$\psi_{k}$	Independent	Frank	Clayton	Gumbel
$100\times \lambda_{AE}$	1	0.2	Frank	1.87	0.288 (0.274, 0.303)	0.206 (0.098, 0.523)	0.283 (0.255, 0.369)	0.270 (0.171, 0.507)
				5.75	0.427 (0.410, 0.445)	0.225 (0.153, 0.483)	0.440 (0.418, 0.482)	0.447 (0.424, 0.513)
			Clayton	0.5	0.063 (0.056, 0.070)	0.064 (0.022, 0.620)	0.207 (0.100, 0.348)	0.531 (0.128, 1.034)
				2	0.002 (0.000, 0.002)	0.000 (0.000, 0.001)	0.045 (0.038, 0.939)	0.001 (0.000, 1.472)
			Gumbel	1.25	0.124 (0.114, 0.133)	0.127 (0.044, 0.689)	0.151 (0.125, 0.245)	0.187 (0.129, 0.555)
				2	0.038 (0.032, 0.044)	0.027 (0.011, 0.196)	0.066 (0.039, 0.182)	0.131 (0.042, 0.611)
	2	1	Frank	1.87	1.325 (1.291, 1.359)	0.998 (0.771, 1.243)	1.263 (1.220, 1.326)	1.024 (0.864, 1.257)
				5.75	1.730 (1.695, 1.768)	1.018 (0.925, 1.218)	1.657 (1.601, 1.710)	1.754 (1.720, 1.792)
			Clayton	0.5	0.581 (0.560, 0.603)	1.476 (1.211, 1.750)	0.984 (0.861, 1.114)	1.549 (1.250, 1.808)
				2	0.092 (0.084, 0.101)	2.303 (0.073, 2.548)	0.982 (0.768, 1.228)	2.077 (1.703, 2.446)
			Gumbel	1.25	0.687 (0.661, 0.711)	0.932 (0.613, 1.200)	0.787 (0.708, 0.901)	0.946 (0.719, 1.257)
				2	0.280 (0.266, 0.295)	0.991 (0.473, 1.485)	0.494 (0.378, 0.612)	0.912 (0.559, 1.251)
	3	5	Frank	1.87	5.451 (5.364, 5.535)	5.029 (4.871, 5.163)	5.474 (5.387, 5.558)	4.948 (3.047, 5.206)
				5.75	5.890 (5.817, 5.964)	5.034 (4.920, 5.170)	5.914 (5.840, 5.990)	5.978 (5.902, 6.056)
			Clayton	0.5	4.401 (4.329, 4.490)	5.050 (4.919, 5.170)	4.991 (4.865, 5.116)	5.109 (4.974, 5.238)
				2	3.028 (2.970, 3.093)	5.110 (4.996, 5.225)	4.971 (4.857, 5.101)	5.180 (5.064, 5.294)
			Gumbel	1.25	4.326 (4.240, 4.407)	4.917 (4.793, 5.045)	4.848 (4.723, 4.970)	5.002 (4.870, 5.130)
				2	3.103 (3.034, 3.177)	4.862 (4.734, 4.978)	4.638 (4.511, 4.759)	4.983 (4.863, 5.100)
CP(NoC)	1	0.95	Frank	1.87	0.021 (21)	0.752 (629)	0.975 (890)	0.880 (863)
				5.75	0.000 (0)	0.828 (749)	0.239 (231)	0.862 (301)
			Clayton	0.5	0.000 (0)	0.525 (264)	0.928 (894)	0.785 (711)
				2	0.000 (0)	0.713 (62)	0.903 (660)	0.900 (242)
			Gumbel	1.25	0.003 (3)	0.666 (411)	0.638 (560)	0.935 (571)
				2	0.000 (0)	0.694 (227)	0.834 (563)	0.898 (538)
	2	0.95	Frank	1.87	0.000 (0)	0.856 (853)	0.937 (921)	0.937 (937)
				5.75	0.000 (0)	0.919 (899)	0.015 (15)	0.046 (9)
			Clayton	0.5	0.000 (0)	0.812 (811)	0.977 (963)	0.000 (0)
				2	0.000 (0)	0.174 (130)	0.962 (962)	0.519 (519)
			Gumbel	1.25	0.000 (0)	0.890 (885)	0.718 (712)	0.935 (662)
				2	0.000 (0)	0.840 (795)	0.306 (302)	0.957 (833)
	3	0.95	Frank	1.87	0.188 (188)	0.992 (990)	0.138 (137)	0.680 (602)
				5.75	0.000 (0)	0.997 (968)	0.002 (2)	0.000 (0)
			Clayton	0.5	0.013 (13)	0.961 (961)	0.978 (978)	0.923 (923)
				2	0.000 (0)	0.815 (815)	0.892 (892)	0.647 (647)
			Gumbel	1.25	0.003 (3)	0.970 (970)	0.947 (947)	0.980 (980)
				2	0.000 (0)	0.861 (861)	0.456 (456)	0.913 (913)
Selected by AIC	1		Frank	1.87	792	101	40	67
				5.75	792	169	31	8
			Clayton	0.5	552	67	196	185
				2	382	51	425	142
			Gumbel	1.25	774	115	59	52
				2	753	80	106	61
	2		Frank	1.87	687	231	5	77
				5.75	271	728	1	0
			Clayton	0.5	182	178	486	154
				2	2	9	740	249
			Gumbel	1.25	742	94	90	74
				2	471	158	256	115
	3		Frank	1.87	157	679	0	164
				5.75	0	1,000	0	0
			Clayton	0.5	16	156	552	276
				2	0	137	832	31
			Gumbel	1.25	50	155	246	549
				2	0	262	74	664

Table 6 presents the summary of the estimates and the coverage probabilities for the hazard parameter with the proposed methods under the independent and dependent follow-up duration assumptions for Dataset 1A. When assuming independence correctly for Dataset 1A, the proposed method had successful convergence for all the simulated dataset (it is given as NoC in Table 6) in maximizing the likelihood function. We found that the bias on $\lambda_{AE}$ was negligible and the empirical CP was close to the nominal level in all scenarios. Although modeling dependence with copula functions is unnecessary for independent data (Dataset 1A), predetermining independence is challenging. Thus, we evaluated performance of the method under the dependent follow-up duration applied to the independent data. We observed that the proposed method sometimes could not obtain estimates in particular for scenarios 1 and 2 and for Frank copula. We also observed that the extra modeling of dependence with the Clayton copula led negligible biases for $\lambda_{AE}$ and provided conservative, but still valid confidence intervals. With the Frank and Gumbel copulas, there were certain biased when the event rate was low (scenario 1) and the empirical coverage probabilities were far from the nominal level except when the event rate was high (scenario 3). Table 6 also shows frequencies that each model was selected with AIC. In approximately 75 % simulated datasets, the independence model was correctly selected across all the scenarios.

The results for Dataset 2A were summarized in Table 7. When the event rate was moderate (scenario 2) or high (scenario 3), the proposed method had only negligible biases on $\lambda_{AE}$ with a correctly-specified copula function. The model with correctly-specified copula provided the empirical coverage probabilities close to the nominal level except for some cases such as Frank with $\psi_{k}$ =1.87 in scenario 2. On the other hand, when the event rate was low (scenario 1), even if the supposed copula model covered the true dependence, there might be certain biases and empirical coverage probabilities away from the nominal level. Table 7 also indicates that misspecified copula modeling of dependence might lead seriously biased estimates for $\lambda_{AE}$ without relevant control of the nominal level for the confidence interval. Frequencies that each model was selected with AIC were presented in Table 7. When the event rate was low (scenario 1) or dependence was low with Knedall’s τ =0.2, the independence model was likely to be selected although the datasets were generated from dependent copulas. If event rate was high (scenario 3), AIC selected the true copula the most frequently.

**Table 8:** Summary of estimates (median (q1,q3)) and empirical coverage probabilities (CP) of the exponential parameter for the time-to-AE and frequencies that each model was selected with AIC for Dataset 1B (*S* = 30, independence): True implies the value of $\lambda_{AE}(\times 100)$ , and NoC is the number of realizations with successful convergence in iteration for the maximum likelihood estimation Table 8 Long description.

Parameter	Scenario	TRUE	Independent	Frank	Clayton	Gumbel
$100\times \lambda_{AE}$	1	0.2	0.200 (0.192, 0.208)	0.210 (0.109, 0.389)	0.209 (0.180, 0.259)	0.227 (0.154, 0.354)
	2	1	0.996 (0.976, 1.018)	0.996 (0.875, 1.120)	0.995 (0.943, 1.071)	1.029 (0.894, 1.170)
	3	5	4.966 (4.902, 5.028)	4.998 (4.917, 5.082)	5.018 (4.944, 5.099)	5.062 (4.988, 5.145)
						
CP(NoC)	1	0.95	0.970 (970)	0.799 (661)	0.983 (894)	0.875 (870)
	2	0.95	0.964 (964)	0.956 (956)	0.989 (974)	0.928 (928)
	3	0.95	0.963 (963)	0.987 (987)	0.978 (963)	0.962 (962)
						
Selected by AIC	1		762	85	47	106
	2		729	76	28	167
	3		762	45	15	179

**Table 9:** Summary of estimates (median (q1,q3)) and empirical coverage probabilities (CP) of the exponential parameter and frequencies that each model was selected with AIC for the time-to-AE for Dataset 2B (*S* = 30, dependence): TRUE implies the value of $\lambda_{AE}(\times 100)$ , dep. shows the applied copula, and NoC is the number of realizations with successful convergence in iteration for the maximum likelihood estimation Table 9 Long description.

Parameter	scn.	TRUE	dep.	$\psi_{k}$	Independent	Frank	Clayton	Gumbel
$100\times \lambda_{AE}$	1	0.2	Frank	1.87	0.287 (0.277, 0.298)	0.233 (0.138, 0.382)	0.274 (0.255, 0.324)	0.276 (0.206, 0.381)
				5.75	0.419 (0.407, 0.431)	0.251 (0.178, 0.364)	0.392 (0.374, 0.421)	0.342 (0.287, 0.419)
			Clayton	0.5	0.071 (0.066, 0.076)	0.303 (0.045, 0.948)	0.196 (0.144, 0.257)	0.473 (0.289, 0.666)
				2	0.002 (0.001, 0.003)	0.001 (0.000, 0.007)	0.239 (0.037, 0.625)	0.474 (0.014, 1.092)
			Gumbel	1.25	0.126 (0.119, 0.132)	0.186 (0.072, 0.429)	0.138 (0.113, 0.183)	0.193 (0.105, 0.349)
				2	0.040 (0.037, 0.044)	0.037 (0.014, 0.220)	0.071 (0.039, 0.120)	0.137 (0.036, 0.340)
	2	1	Frank	1.87	1.294 (1.271, 1.318)	1.015 (0.895, 1.127)	1.236 (1.206, 1.274)	1.089 (1.002, 1.186)
				5.75	1.654 (1.630, 1.678)	1.018 (0.950, 1.116)	1.600 (1.564, 1.627)	1.223 (1.149, 1.295)
			Clayton	0.5	0.624 (0.609, 0.639)	1.265 (1.153, 1.380)	0.978 (0.913, 1.051)	1.377 (1.246, 1.498)
				2	0.138 (0.131, 0.144)	2.205 (2.006, 2.371)	0.984 (0.881, 1.074)	1.760 (1.628, 1.889)
			Gumbel	1.25	0.703 (0.686, 0.720)	0.937 (0.805, 1.057)	0.821 (0.747, 0.886)	0.989 (0.834, 1.117)
				2	0.306 (0.296, 0.318)	0.943 (0.767, 1.121)	0.545 (0.472, 0.615)	0.931 (0.757, 1.088)
	3	5	Frank	1.87	5.283 (5.225, 5.345)	5.011 (4.923, 5.091)	5.472 (5.398, 5.576)	5.101 (5.017, 5.182)
				5.75	5.573 (5.517, 5.631)	5.011 (4.931, 5.103)	5.011 (4.931, 5.103)	5.143 (5.051, 5.234)
			Clayton	0.5	4.568 (4.509, 4.627)	5.006 (4.932, 5.078)	5.155 (4.989, 7.825)	5.053 (4.981, 5.126)
				2	3.586 (3.532, 3.633)	5.001 (4.937, 5.066)	5.006 (4.938, 5.074)	5.018 (4.954, 5.083)
			Gumbel	1.25	4.519 (4.456, 4.579)	4.999 (4.920, 5.065)	5.113 (4.965, 7.672)	5.049 (4.969, 5.113)
				2	3.664 (3.610, 3.723)	5.001 (4.938, 5.065)	4.987 (4.921, 5.055)	5.029 (4.967, 5.090)
CP(NoC)	1	0.95	Frank	1.87	0.000 (0)	0.859 (801)	0.932 (838)	0.955 (953)
				5.75	0.000 (0)	0.899 (881)	0.377 (320)	0.973 (973)
			Clayton	0.5	0.000 (0)	0.608 (454)	0.976 (973)	0.809 (781)
				2	0.000 (0)	0.622 (97)	0.924 (645)	0.815 (564)
			Gumbel	1.25	0.000 (0)	0.760 (567)	0.942 (853)	0.826 (796)
				2	0.000 (0)	0.564 (285)	0.770 (689)	0.760 (600)
	2	0.95	Frank	1.87	0.000 (0)	0.938 (938)	0.646 (629)	0.976 (976)
				5.75	0.000 (0)	0.930 (920)	0.000 (0)	0.835 (833)
			Clayton	0.5	0.000 (0)	0.644 (644)	0.954 (954)	0.494 (494)
				2	0.000 (0)	0.019 (15)	0.933 (933)	0.032 (32)
			Gumbel	1.25	0.000 (0)	0.963 (963)	0.603 (602)	0.937 (937)
				2	0.000 (0)	0.945 (936)	0.015 (15)	0.940 (940)
	3	0.95	Frank	1.87	0.260 (260)	0.996 (996)	0.018 (17)	0.962 (962)
				5.75	0.000 (0)	0.992 (968)	0.992 (968)	0.954 (954)
			Clayton	0.5	0.019 (19)	0.969 (969)	0.910 (518)	0.946 (946)
				2	0.000 (0)	0.853 (853)	0.834 (834)	0.848 (848)
			Gumbel	1.25	0.001 (1)	0.963 (963)	0.938 (546)	0.943 (924)
				2	0.000 (0)	0.891 (891)	0.880 (880)	0.875 (875)
Selected by AIC	1		Frank	1.87	783	90	15	111
				5.75	656	218	10	116
			Clayton	0.5	278	104	426	192
				2	435	41	328	203
			Gumbel	1.25	724	107	50	119
				2	624	121	130	125
	2		Frank	1.87	334	485	7	174
				5.75	4	946	4	50
			Clayton	0.5	8	136	617	239
				2	0	1	908	91
			Gumbel	1.25	472	115	138	275
				2	81	239	436	244
	3		Frank	1.87	2	701	0	297
				5.75	0	871	0	129
			Clayton	0.5	0	207	478	315
				2	0	200	775	25
			Gumbel	1.25	0	274	69	657
				2	0	157	3	840

The results under the independent follow-up duration with *S* = 30 (Dataset 1B) are presented in Table 8. If the independent copula is correctly applied, the proposed method had negligible biases and empirical coverage probabilities close to the nominal level similarly to the case of *S* = 13 (Table 6). When dependent copulas were applied, increasing the number of studies led substantial reduction of biases. The empirical coverage probabilities might be anti-conservative for a rare event case (scenario 1) even with *S* = 30; when dependence was supposed with Frank and Gumble copulas to the dataset under independence, empirical coverage probabilities were far from the nominal level and anti-conservative. For scenarios 2 and 3, our methods had empirical coverage probabilities close to the nominal level. The results under the dependent follow-up duration with *S* = 30 (Dataset 2B) are presented in Table 9. The proposed method had negligible biases when the copula was correctly specified. With a larger number of studies, interquartile ranges of estimates for $\lambda_{E}$ became shorter. When the event rate was low (scenario 1), empirical coverage probabilities might be far from the nominal level. Overall, the proposed method had empirical coverage probabilities close to the nominal level with the correctly specified copula.

In Table 10, we gave a summary of estimates of the exponential hazard parameter for the censoring distribution of each study. Recall that the at-risk data were available at 6, 12, and 18 months for all the studies in Case 1 and at randomly selected two time points in Case 2. In both cases, all the exponential parameters were appropriately estimated. It suggests that estimation of the censoring distribution with the method in Section 3.2 is not sensitive to what time points are utilized.

## 6 Discussion

Since very limited safety assessment can be made in a single clinical trial, meta-analysis would be a very powerful tool to clarify the safety profile of drugs. An FDA Guidance for Industry^7^ argues various issues in meta-analysis of AEs in randomized clinical trials. The issue of heterogeneous follow-up duration is repeatedly emphasized (see Section III-D in the guidance). Importance of careful consideration of eligibility criterion in study selection is emphasized; without subject-level data, some trials may need to be excluded in the presence of heterogeneous follow-up duration. However, no specific statistical method is mentioned in the guidance to handle the issue except for recommending use of the IPD if possible. To our best knowledge, as of this moment, no established methodologies exist for literature-based meta-analysis of AEs that accounts for heterogeneous follow-up and our proposed method is the first one to handle this situation.

The idea of our proposed method is to utilize the Kaplan–Meier estimates of the primary time-to-event efficacy endpoints as information of the follow-up duration. Displaying the Kaplan–Meier estimates of the OS or the PFS is routinely used in oncology clinical trials. Then, our method is of particularly practical benefit in oncology clinical trials, in which the heterogeneous follow-up duration often matters in the nature of the study design. In oncology clinical trials, the Kaplan–Meier estimates for both of the OS and the PFS may be reported. Note that our idea is to obtain the distribution of the end of follow-up of AEs with them, then we should choose the endpoint (the OS or the PFS) defining the end of the follow-up in the study protocol. The follow-up duration for the AE is determined by other censoring, such as the treatment discontinuation due to some safety issues or lack of efficacy. In most oncology clinical trials, the subject is off the study once the follow-up of the efficacy endpoint is stopped. Then, such subjects are regarded as censored in evaluating the primary time-to-event endpoint. Since the distribution of the follow-up duration was determined by the distribution of the efficacy endpoint and that of the censoring as seen in Section 3.1, the treatment discontinuation is appropriately handled in the proposed method. Although we focus on oncology in this manuscript, there are some other therapeutic areas of potential application of the proposed method, possessing the features discussed in this paragraph, such as cardiovascular disease.

**Table 10:** Summary of the estimates of the study-specific exponential parameter for the censoring distribution Table 10 Long description.

Study	True	Estimated exponential hazard for censoring: median (q1,q3)	
		Case 1	Case 2
1	0.02	0.0201 (0.0178, 0.0223)	0.0197 (0.0178, 0.0221)
2	0.015	0.0150 (0.0136, 0.0163)	0.0150 (0.0138, 0.0162)
3	0.015	0.0150 (0.0137, 0.0162)	0.0149 (0.0133, 0.0167)
4	0.025	0.0249 (0.0229, 0.0270)	0.0246 (0.0224, 0.0265)
5	0.01	0.0092 (0.0060, 0.0131)	0.0068 (0.0016, 0.0115)
6	0.01	0.0099 (0.0082, 0.0118)	0.0099 (0.0083, 0.0115)
7	0.018	0.0175 (0.0145, 0.0214)	0.0180 (0.0152, 0.0209)
8	0.02	0.0199 (0.0184, 0.0215)	0.0200 (0.0181, 0.0221)
9	0.012	0.0118 (0.0088, 0.0155)	0.0117 (0.0092, 0.0147)
10	0.01	0.0096 (0.0072, 0.0123)	0.0101 (0.0081, 0.0120)
11	0.024	0.0240 (0.0218, 0.0261)	0.0240 (0.0221, 0.0263)
12	0.015	0.0150 (0.0140, 0.0160)	0.0150 (0.0139, 0.0161)
13	0.01	0.0098 (0.0084, 0.0115)	0.0101 (0.0086, 0.0117)

Through numerical studies, we found that our method works well if parametric models are appropriately employed. If misspecified, the estimate may not be obtained due to failure in iteration steps for the maximum likelihood estimation and even if the estimate is successfully obtained, it may be biased. When the independence is satisfied, the extra modeling of the dependence might lead poor performance when the number of studies was small (*S* = 13). In our simulation study, we observed that the Clayton copula does not lead any invalid inference, whereas with the Frank and Gumbel copulas, the proposed methods might suffer from bias in estimation, poor control of the nominal level of the confidence interval and non-convergence in iteration steps for estimation probably due to difficulty to handle extra parameters in dependence with limited information from summary statistic data. When the number of studies was large (*S* = 30), the performance of the model improved; the extra copula modeling of dependence did not lead serious deficiencies.

Here, we summarize the steps and the points to be taken care of in applying our proposed method. Recall our proposed method for meta-analysis occurs within treatment groups. To make a valid comparison, the subjects’ characteristics should be similar between the groups and then, careful consideration is needed on inclusion of studies. To apply the proposed method, we need frequencies of the AE of each study and the survival functions of the end of the follow-up for the AE. The latter can be obtained from a graphical plot of the Kaplan–Meier estimate for the time-to-event efficacy primary endpoint (OS or PFS). To estimate the censoring distribution for the efficacy endpoint, information on the number of at-risk, which is often attached below the graphical plot of the Kaplan–Meier estimates, should be obtained (see Table 2). The key quantity to summarize the occurrence of the AE is the cumulative distribution function of the time to occurrence of the AE. Key steps to apply the method are as follows:

1. Specify parametric models for the distribution of the time to occurrence of the AE, $F_{AE,k}(t;\theta_{k})$ .

2. Specify a parametric model for the censoring distribution $G_{k}^{\left(s\right)}(t;\eta_{k})$ and estimate the unknown parameter $\eta_{k}$ with the method in Section 3.2.

3. Specify copula families $C_{k,\psi_{k}}(u,v)$ for the dependence between the time to occurrence of the AE and the duration of the follow-up for the AE.

4. Estimate the unknown parameter $\theta_{k}$ with the method in Section 3.1 under the assumption of the independent follow-up duration. Estimate $\theta_{k}$ , as well as the unknown parameters of the copula, with the method in Section 3.3 under the dependent follow-up duration.

5. Select the best combination of the models for the cumulative distribution function for the time to occurrence of the AE and that for the copula attaining the minimum AIC value. The goodness-of-fit can be evaluated by the graphical plots of the observed and model-based incidence proportions with the chi-squared goodness-of-fit test as illustrated in Section 4.

6. Present the cumulative distribution functions $F_{AE:k}(t;{\hat{\theta }}_{k})$ and their RR at several time points as done in Figures 2 and 4 and Table 4.

One may apply the Poisson regression model with the follow-up durations of each study as the offset term if the follow-up duration is available for all the studies. This was suggested by Cai et al.,^4^ although they did not discuss how to determine the follow-up duration in practice. It can be obtained with Kaplan–Meier estimate of the efficacy endpoint and the censoring distribution with the method discussed in this article. The Poisson regression assumes the exponential distribution for the time-to-AE and then our method can handle more flexible class of the distributions for the AE.

The motivation of this research came from difficulty in interpretation of the incidence proportion in the presence of heterogeneous follow-up duration. However, we do not criticize importance of the incidence proportion in practice. It is very useful to capture overall tendency in safety profiles of drugs. On the other hand, when one need to make delicate evaluation of the usefulness of drugs considering balance between efficacy and risk of AEs, it is not appealing to simply compare incidence proportions among the treatments. In oncology clinical trials, the follow-up duration is often restricted by the time-to-event efficacy endpoint and then superiority with respect to the primary endpoint leads longer follow-up duration of AEs. Thus, the drug of superior efficacy may have overly estimated incidence proportions of AEs. To compare the risk and benefit of drugs, proper adjustment of the heterogeneous follow-up durations should be considered and the proposed method would be useful in such situations.

In our empirical data analysis, the precision of fit was compromised due to the inclusion of trials with no AEs in the control group. In the simulation study, we also observed that our proposed method might not work well for AEs of a low event rate. However, our method worked well for AEs of a certainly high event rate. In addressing the risk and benefit of drugs, we usually focus on commonly observed AEs. In such situations, our method worked well and would be useful in practice.

On the other hand, one may need to compare rare AEs among several drugs and the issue of heterogeneous follow-up durations can also have serious impacts on the comparison. Then, it is important to extend our inference procedure so as to provide stable estimates for rare events. To achieve this, it will be necessary to explore extensions, such as Bayesian estimation or penalized inference methods, which we consider as future research topics.

A limitation of our method is it assumes the common cumulative incidence function for the AE over studies. It corresponds to the fixed-effect model in meta-analysis. On the other hand, one may hope to evaluate heterogeneity of occurrence of AEs over studies and then the mixed-effect models would be more appealing. It would warrant to develop inference procedures to incorporate random effects to evaluate heterogeneity. In the proposed method, we fit the model and then estimate the cumulative incidence over time separately within the groups. It allows to fit difference parametric models to each group flexibly. On the other hand, it also implies that outcomes from the same randomized study may be analyzed compromising randomization. This issue is argued in the context of network meta-analysis. In the standard pairwise meta-analysis of randomized trials, a treatment contrast, such as the log-transformed odds ratio between the two treatment groups, is used as the outcome for meta-analysis respecting randomization. This approach is called the contrast-based approach since the outcome is the treatment contrast estimated within a study. In network meta-analysis, studies of various combinations of treatments are included and then background characteristics may not be homogeneous over the treatment groups to compare even if the contrast-based approach is taken. Alternatively, the arm-based approach is of interest and its pros and cons were discussed; White et al.^19^ reported that the arm-based approach may lead to more biased results than the contrast-based one. Our method corresponds to the arm-based method and then studies included in meta-analysis should be carefully determined. An approach is to include only randomized studies with both treatment groups to be compared in meta-analysis or studies with only either group are excluded, respecting randomization. On the other hand, it is desirable to include a wider range of studies in meta-analysis. To this end, it is important to develop methods for adjusting confounders, possibly through regression modeling, for example, for $\theta_{k}$ in $F_{AE:k}(t;\theta_{k})$ , that incorporate Bayesian estimation and penalized inference techniques to ensure stable implementation.

Same as for efficacy evaluation, the publication bias may be a serious threat in meta-analysis of AEs.^20^ ^–^ ^22^ For the proposed method, we need to consider other type of reporting bias. Our method requires the Kaplan–Meier estimates for the efficacy time-to-event endpoints and they may not be reported; as explained in our example, 5 studies among 17 studies did not report the Kaplan–Meier estimates and then they were excluded in our meta-analysis. It is very valuable to develop methods to address potential biases due to such kind of selective sampling in our proposed method.

## Data Availability

The dataset was created using a graph scan software with a dataset available to the public. It is available at 10.5281/zenodo.16872253 with an SAS code to implement the proposed method.

## Long descriptions

### Table 1 Long description

The table contains 17 rows of study data with the following columns: No., Study I D, n (number of subjects), M F T (median follow-up time in months), I P (incidence proportion) for bev. (bevacizumab) and cnt. (control) groups, Primary endpoint, Follow-up definition, K M (Kaplan-Meier availability), and At-risk information availability.

* Row 1: Aghajanian 2012; n 480; M F T 24; I P bev. 0.81%; I P cnt. 0.43%; Primary P F S; Follow-up P F S plus 1; K M and At-risk reported.

* Row 2: Allegra 2009; n 2,647; M F T 28.5; I P bev. 0.38%; I P cnt. 0.38%; Primary A E incidence; Follow-up N A; K M and At-risk missing.

* Row 3: Burger 2011; n 1,209; M F T 17.4; I P bev. 0.33%; I P cnt. 0.00%; Primary P F S; Follow-up P F S plus 1; K M and At-risk reported.

* Row 4: Chinot 2014; n 921; M F T N A; I P bev. 1.95%; I P cnt. 0.89%; Primary O S, P F S; Follow-up P F S plus 3; K M and At-risk reported.

* Row 5: Giantonio 2007; n 572; M F T 28; I P bev. 1.04%; I P cnt. 0.00%; Primary O S; Follow-up O S; K M reported; At-risk missing.

* Row 6: Herbst 2011; n 626; M F T 19; I P bev. 0.32%; I P cnt. 0.00%; Primary O S; Follow-up O S; K M and At-risk reported.

* Row 7: Kabbinavar 2003; n 102; M F T 17.6; I P bev. 6.25%; I P cnt. 0.00%; Primary P F S; Follow-up N A; K M and At-risk missing.

* Row 8: Kelly 2012; n 1,009; M F T 25; I P bev. 1.39%; I P cnt. 0.20%; Primary O S; Follow-up P F S plus 1; K M and At-risk reported.

* Row 9: Kim 2012; n 212; M F T 13; I P bev. 0.00%; I P cnt. 0.00%; Primary P F S; Follow-up P F S plus 1; K M and At-risk reported.

* Row 10: Kindler 2007; n 108; M F T 15.6; I P bev. 3.77%; I P cnt. 0.00%; Primary P F S; Follow-up N A; K M and At-risk missing.

* Row 11: Kindler 2010; n 540; M F T N A; I P bev. 0.72%; I P cnt. 0.76%; Primary O S; Follow-up O S; K M reported; At-risk missing.

* Row 12: Miller 2007; n 711; M F T 41.6; I P bev. 1.92%; I P cnt. 0.00%; Primary P F S; Follow-up O S; K M and At-risk reported.

* Row 13: Okines 2013; n 200; M F T 22; I P bev. 1.01%; I P cnt. 0.00%; Primary G I perforation rates; Follow-up N A; K M and At-risk missing.

* Row 14: Perren 2011; n 1,528; M F T N A; I P bev. 0.27%; I P cnt. 0.00%; Primary O S, P F S; Follow-up P F S plus 1.5; K M and At-risk reported.

* Row 15: Price 2012; n 471; M F T 30.8; I P bev. 1.27%; I P cnt. 0.00%; Primary O S, P F S; Follow-up N A; K M and At-risk missing.

* Row 16: Rini 2010; n 709; M F T N A; I P bev. 1.38%; I P cnt. 0.29%; Primary O S; Follow-up O S; K M and At-risk reported.

* Row 17: Sandler 2006; n 867; M F T 19; I P bev. 1.17%; I P cnt. 0.00%; Primary O S; Follow-up O S; K M reported; At-risk missing.

Navigate back to Table 1.

### Table 2 Long description

The table is organized into three columns: Index, Study/Treatment/Subject, and Notation/Definition.

* Index Section:

- s: study where s equals 1, 2, through S.

- k: treatment group where 1 is experimental and 0 is control.

- i: subject.

* Time-to-event variable Section:

- T sub k,i super (s): time to primary endpoint (O S or P F S).

- C sub k,i super (s): potential censoring time for T sub k,i super (s).

- T-tilde sub k,i super (s) = min (T sub k,i super (s), C sub k,i super (s)).

- T sub A E: k,i super (s): time to A E.

- T sub F U: k,i super (s): follow-up duration for A E.

- T-tilde sub F U: k,i super (s) = min (T sub F U: k,i super (s), C sub k,i super (s)).

* Function Section:

- S sub k super (s) (t) = P (T sub k,i super (s) > t).

- F sub k super (s) (t) = 1 minus S sub k super (s) (t).

- G sub k super (s) (t) = P (C sub k,i super (s) > t).

- S sub F U: k super (s) (t) = P (T sub F U: k,i super (s) > t).

- F sub F U: k super (s) (t) = 1 minus S sub F U: k super (s) (t).

- S-tilde sub F U: k super (s) (t) = P (T-tilde sub F U: k,i super (s) > t).

- F-tilde sub F U: k super (s) (t) = 1 minus S-tilde sub F U: k super (s) (t).

- S sub A E: k (t) = P (T sub A E: k,i > t).

- F sub A E: k (t) = 1 minus S sub A E: k (t).

* Observation Section:

- S-hat sub k super (s) (t): Kaplan-Meier estimate for S sub k super (s) (t).

- n sub k super (s): the number of subjects.

- X sub k super (s): the number of A Es.

- a sub k super (s): the number of subjects at-risk at time t.

Navigate back to Table 2.

### Table 3 Long description

The table is structured with two main header levels. The top level divides the data into Bevacizumab group and Control group. The second level specifies three distribution models for each group: Exponential, Weibull, and Log-normal. The rows are categorized by Follow-up type and Copula.

* Independent Follow-up with Independent Copula:

- Bevacizumab group: Exponential 62.3, Weibull 60.7, Log-normal 60.7.

- Control group: Exponential 34.2, Weibull N A, Log-normal N A.

* Dependent Follow-up:

- Clayton Copula: Bevacizumab (63.4, 63.4, 63.5); Control (36.0, N A, 35.6).

- Frank Copula: Bevacizumab (64.0, 62.4, 62.1); Control (35.1, N A, N A).

- Gumbel Copula: Bevacizumab (64.4, 62.1, 61.6); Control (33.9, 35.9, 45.7).

N A indicates non-convergence of non-linear optimization.

Navigate back to Table 3.

### Figure 1 Long description

A two-panel scatter plot. Both panels feature a horizontal x-axis labeled Crude incidence proportion and a vertical y-axis labeled Model-based incidence proportion. A diagonal line of identity (y equals x) bisects each graph from the bottom-left origin to the top-right corner.

Left Panel (Bevacizumab Group):

* The x-axis and y-axis both range from 0.000 to 0.030.

* Text in the top-left corner reads: Time-to-A E: log-normal; Copula: independent; A I C = 60.3; p-value = 0.021.

* Data points are represented by open circles. Most points cluster near the origin between 0.000 and 0.010. Several points are positioned above the diagonal line, indicating the model slightly overestimates incidence compared to crude data in those instances.

Right Panel (Control Group):

* The x-axis and y-axis both range from 0.000 to 0.012.

* Text in the top-left corner reads: Time-to-A E: exponential; Copula: independent; A I C = 34.2; p-value = 0.012.

* Data points are represented by open circles. A dense vertical cluster of points appears at the x-axis value of 0.000. Other points are scattered below the diagonal line, suggesting the model underestimates incidence for those specific observations.

Navigate back to Figure 1.

### Figure 2 Long description

The figure consists of two side-by-side line graphs.

Left Panel: Cumulative incidence rate.

* The x-axis is labeled Month, ranging from 0 to 40. The y-axis is labeled Cumulative incidence rate, ranging from 0.00 to 0.06.

* The bevacizumab group is represented by a solid red curve that shows an exponential-like increase, reaching approximately 0.04 at 40 months. It is flanked by two dashed red curves with open circles representing the 95 percent confidence bands.

* The control group is represented by a solid blue curve that shows a much flatter, slight linear increase, reaching just below 0.01 at 40 months. It is also flanked by dashed blue curves with open circles for confidence bands.

Right Panel: Hazard functions.

* The x-axis is labeled Month, ranging from 0 to 40. The y-axis is labeled Hazard, ranging from 0.000 to 0.004.

* The bevacizumab group is shown as a red curve that rises sharply from 0 and then begins to plateau, reaching a hazard of approximately 0.0015 by month 40.

* The control group is shown as a flat, horizontal blue line constant at a hazard level very close to 0.0001 throughout the 40-month period.

Navigate back to Figure 2.

### Figure 3 Long description

A two-panel scatter plot. Both panels feature a horizontal x-axis labeled Crude incedence proportion and a vertical y-axis labeled Model-based incedence proportion. A diagonal line representing a perfect one-to-one fit bisects each graph from the origin.

Left Panel (Bevacizumab Group):

* X and Y axes range from 0.000 to 0.030.

* Text in the top-left corner: Time-to-A E: log-normal, Copula: Gumbel, A I C = 61.6, p-value = 0.030.

* Data points: Approximately 13 open circles are scattered around the diagonal line. Most points are clustered between 0.000 and 0.015 on both axes, with one outlier near x = 0.014, y = 0.023.

Right Panel (Control Group):

* X and Y axes range from 0.000 to 0.012.

* Text in the top-left corner: Time-to-A E: exponential, Copula: Gumbel, A I C = 33.9, p-value = 0.013.

* Data points: Approximately 13 open circles. A dense vertical cluster of points is located at x = 0.000, ranging from y = 0.000 to 0.002. Other points are scattered below the diagonal line as the crude incidence increases toward 0.009.

Navigate back to Figure 3.

### Figure 4 Long description

A two-panel line graph.

Left Panel: Cumulative incidence rate.

- The x-axis is labeled Month, ranging from 0 to 40.

- The y-axis is labeled Cumulative incidence rate, ranging from 0.00 to 0.06.

- The bevacizumab group is represented by a solid red curve that shows an exponential-like increase, reaching approximately 0.033 at 40 months. It is flanked by two dashed red curves representing the 95 percent confidence bands, which widen significantly as time progresses.

- The control group is represented by a solid blue curve that shows a much slower, nearly linear increase, reaching approximately 0.005 at 40 months. Its 95 percent confidence bands, shown as dashed blue lines, remain much tighter than the bevacizumab group.

Right Panel: Hazard.

- The x-axis is labeled Month, ranging from 0 to 40.

- The y-axis is labeled Hazard, ranging from 0.000 to 0.004.

- The bevacizumab group is shown as a red curve that starts at 0, rises sharply until month 20, and then begins to plateau, reaching approximately 0.0015 at 40 months.

- The control group is shown as a flat, horizontal blue line at a constant hazard level of approximately 0.0002 across the entire 40-month duration.

Navigate back to Figure 4.

### Table 4 Long description

The table consists of five columns. The first column is time t, with values 5, 10, 15, 20, 25, 30, 35, and 40. The next two columns fall under the Independent category, providing R R (95 percent C I) and P-value. The final two columns fall under the Dependent category, providing R R (95 percent C I) and P-value.

* At t = 5: Independent R R is 1.404 (0.134, 14.738), P-value 0.777. Dependent R R is 0.394 (0.010, 15.183), P-value 0.617.

* At t = 10: Independent R R is 3.178 (0.860, 11.739), P-value 0.083. Dependent R R is 1.289 (0.118, 14.089), P-value 0.835.

* At t = 15: Independent R R is 4.664 (1.835, 11.854), P-value 0.001. Dependent R R is 2.273 (0.331, 15.633), P-value 0.404.

* At t = 20: Independent R R is 5.875 (2.581, 13.377), P-value less than 0.001. Dependent R R is 3.215 (0.566, 18.271), P-value 0.188.

* At t = 25: Independent R R is 6.865 (2.998, 15.720), P-value less than 0.001. Dependent R R is 4.077 (0.772, 21.530), P-value 0.098.

* At t = 30: Independent R R is 7.679 (3.197, 18.443), P-value less than 0.001. Dependent R R is 4.851 (0.936, 25.128), P-value 0.060.

* At t = 35: Independent R R is 8.354 (3.282, 21.268), P-value less than 0.001. Dependent R R is 5.540 (1.063, 28.870), P-value 0.042.

* At t = 40: Independent R R is 8.918 (3.308, 24.041), P-value less than 0.001. Dependent R R is 6.153 (1.160, 32.619), P-value 0.033.

Navigate back to Table 4.

### Table 5 Long description

The table consists of 6 columns: study, Authors, n, Median P F S, lambda super (s), and lambda sub C super (s).

* Study 1: Aghajanian, 2012; n = 242; Median P F S = 12.4; lambda super (s) = 0.0243; lambda sub C super (s) = 0.02.

* Study 3-1: Burger, 2011; n = 625; Median P F S = 11.2; lambda super (s) = 0.0269; lambda sub C super (s) = 0.015.

* Study 3-2: Burger, 2011; n = 623; Median P F S = 14.1; lambda super (s) = 0.0213; lambda sub C super (s) = 0.015.

* Study 4: Chinot, 2014; n = 458; Median P F S = 10.6; lambda super (s) = 0.0284; lambda sub C super (s) = 0.025.

* Study 5-1: Giantonio, 2007; n = 237; Median P F S = 2.7; lambda super (s) = 0.1115; lambda sub C super (s) = 0.01.

* Study 5-2: Giantonio, 2007; n = 280; Median P F S = 7.3; lambda super (s) = 0.0412; lambda sub C super (s) = 0.015.

* Study 6: Herbst, 2011; n = 319; Median P F S = 3.4; lambda super (s) = 0.0885; lambda sub C super (s) = 0.018.

* Study 8: Kelly, 2012; n = 524; Median P F S = 9.9; lambda super (s) = 0.0304; lambda sub C super (s) = 0.02.

* Study 9: Kim, 2012; n = 143; Median P F S = 5.6; lambda super (s) = 0.0538; lambda sub C super (s) = 0.012.

* Study 11: Kindler, 2010; n = 300; Median P F S = 3.8; lambda super (s) = 0.0792; lambda sub C super (s) = 0.01.

* Study 12: Miller, 2007; n = 347; Median P F S = 11.8; lambda super (s) = 0.0255; lambda sub C super (s) = 0.024.

* Study 14: Perren, 2011; n = 764; Median P F S = 19.8; lambda super (s) = 0.0152; lambda sub C super (s) = 0.015.

* Study 17: Sandler, 2006; n = 417; Median P F S = 6.2; lambda super (s) = 0.0486; lambda sub C super (s) = 0.01.

Navigate back to Table 5.

### Table 6 Long description

The table is structured with two main header groups: Data (Scenario and True value) and Model (Independent, Frank, Clayton, and Gumbel). It is divided into three vertical sections based on the Parameter.

1. Parameter 100 times lambda sub A E (median and quartiles):

* Scenario 1 (True 0.2): Independent 0.199 (0.187, 0.212); Frank 0.158 (0.068, 0.582); Clayton 0.224 (0.199, 0.302); Gumbel 0.238 (0.203, 0.518).

* Scenario 2 (True 1): Independent 0.998 (0.968, 1.026); Frank 1.006 (0.739, 1.267); Clayton 1.038 (0.993, 1.134); Gumbel 1.060 (1.006, 1.317).

* Scenario 3 (True 5): Independent 4.980 (4.899, 5.062); Frank 4.994 (4.843, 5.139); Clayton 5.041 (4.946, 5.153); Gumbel 5.102 (5.014, 5.216).

2. Parameter C P (N o C) (Empirical coverage probability and number of successful realizations):

* Scenario 1 (True 0.95): Independent 0.960 (960); Frank 0.735 (546); Clayton 0.976 (911); Gumbel 0.974 (526).

* Scenario 2 (True 0.95): Independent 0.975 (975); Frank 0.890 (884); Clayton 0.976 (959); Gumbel 0.948 (494).

* Scenario 3 (True 0.95): Independent 0.983 (983); Frank 0.987 (987); Clayton 0.977 (959); Gumbel 0.969 (818).

3. Parameter Selected by A I C (Frequency of model selection):

* Scenario 1: Independent 790; Frank 144; Clayton 39; Gumbel 27.

* Scenario 2: Independent 832; Frank 91; Clayton 38; Gumbel 39.

* Scenario 3: Independent 857; Frank 68; Clayton 26; Gumbel 49.

Navigate back to Table 6.

### Table 7 Long description

The table is divided into three primary vertical sections based on the Parameter column.

1. 100 times lambda sub A E (Exponential Parameter Estimates):

- Scenario 1 (True value 0.2): Median estimates range from 0.000 to 0.531. The Frank model under Frank dependency yields 0.206 (0.098, 0.523).

- Scenario 2 (True value 1): Median estimates range from 0.092 to 2.303. The Clayton model under Clayton dependency yields 0.984 (0.861, 1.114).

- Scenario 3 (True value 5): Median estimates range from 3.028 to 5.978. The Gumbel model under Gumbel dependency yields 5.002 (4.870, 5.130) at psi sub k equals 1.25.

2. C P (N o C) (Coverage Probabilities and Successful Convergences):

- Scenario 1: Coverage probabilities for the true model type generally range from 0.638 to 0.975. Successful convergences (N o C) vary widely, with the Clayton model reaching 894 under Clayton dependency.

- Scenario 2: Frank model under Frank dependency shows C P of 0.856 (853 convergences) at psi sub k equals 1.87.

- Scenario 3: Gumbel model under Gumbel dependency shows C P of 0.980 (980 convergences) at psi sub k equals 1.25.

3. Selected by A I C (Frequency of model selection):

- Scenario 1: The Independent model is frequently selected (up to 792 times) even when dependency exists.

- Scenario 2: Under Frank dependency (psi sub k equals 5.75), the Frank model is selected 728 times.

- Scenario 3: Under Clayton dependency (psi sub k equals 2), the Clayton model is selected 832 times. Under Gumbel dependency (psi sub k equals 2), the Gumbel model is selected 664 times.

Navigate back to Table 7.

### Table 8 Long description

The table consists of 7 columns: Parameter, Scenario, T R U E, Independent, Frank, Clayton, and Gumbel. It is divided into three vertical sections based on the Parameter.

1. Parameter 100 times lambda sub A E (median and quartiles):

* Scenario 1 (TRUE 0.2): Independent 0.200 (0.192, 0.208); Frank 0.210 (0.109, 0.389); Clayton 0.209 (0.180, 0.259); Gumbel 0.227 (0.154, 0.354).

* Scenario 2 (TRUE 1): Independent 0.996 (0.976, 1.018); Frank 0.996 (0.875, 1.120); Clayton 0.995 (0.943, 1.071); Gumbel 1.029 (0.894, 1.170).

* Scenario 3 (TRUE 5): Independent 4.966 (4.902, 5.028); Frank 4.998 (4.917, 5.082); Clayton 5.018 (4.944, 5.099); Gumbel 5.062 (4.988, 5.145).

2. Parameter C P (N o C) (Coverage Probability and Number of Convergences):

* Scenario 1 (TRUE 0.95): Independent 0.970 (970); Frank 0.799 (661); Clayton 0.983 (894); Gumbel 0.875 (870).

* Scenario 2 (TRUE 0.95): Independent 0.964 (964); Frank 0.956 (956); Clayton 0.989 (974); Gumbel 0.928 (928).

* Scenario 3 (TRUE 0.95): Independent 0.963 (963); Frank 0.987 (987); Clayton 0.978 (963); Gumbel 0.962 (962).

3. Parameter Selected by A I C (Frequency of model selection):

* Scenario 1: Independent 762; Frank 85; Clayton 47; Gumbel 106.

* Scenario 2: Independent 729; Frank 76; Clayton 28; Gumbel 167.

* Scenario 3: Independent 762; Frank 45; Clayton 15; Gumbel 179.

Navigate back to Table 8.

### Table 9 Long description

The table is divided into three primary horizontal sections based on the Parameter column.

1. Section 1: 100 times lambda sub A E (Exponential Parameter Estimates)

- Scenario 1 (True 0.2): For Frank dependence (psi sub k 1.87), the Frank model estimate is 0.233 (0.138, 0.382). For Clayton dependence (psi sub k 0.5), the Clayton model estimate is 0.196 (0.144, 0.257).

- Scenario 2 (True 1): For Frank dependence (psi sub k 5.75), the Frank model estimate is 1.018 (0.950, 1.116). For Gumbel dependence (psi sub k 2), the Gumbel model estimate is 0.931 (0.757, 1.088).

- Scenario 3 (True 5): For Clayton dependence (psi sub k 2), the Clayton model estimate is 5.006 (4.938, 5.074).

2. Section 2: C P open parenthesis N o C close parenthesis (Empirical Coverage Probabilities and Convergence Counts)

- Scenario 1: Independent models consistently show 0.000 (0). Under Frank dependence (psi sub k 5.75), the Gumbel model shows a C P of 0.973 with 973 successful realizations.

- Scenario 2: Under Clayton dependence (psi sub k 2), the Clayton model shows a C P of 0.933 (933 realizations).

- Scenario 3: Under Gumbel dependence (psi sub k 2), the Gumbel model shows a C P of 0.875 (875 realizations).

3. Section 3: Selected by A I C (Frequency of Model Selection)

- Scenario 1: When the true dependence is Clayton (psi sub k 0.5), the Clayton model is selected 426 times, while the Independent model is selected 278 times.

- Scenario 2: When the true dependence is Frank (psi sub k 5.75), the Frank model is selected 946 times.

- Scenario 3: When the true dependence is Gumbel (psi sub k 2), the Gumbel model is selected 840 times.

Navigate back to Table 9.

### Table 10 Long description

The table consists of 13 rows of data under four main column headers: Study, True, Case 1, and Case 2. The Case 1 and Case 2 columns represent the estimated exponential hazard for censoring, reported as median (q 1, q 3).

* Study 1: True 0.02; Case 1 0.0201 (0.0178, 0.0223); Case 2 0.0197 (0.0178, 0.0221).

* Study 2: True 0.015; Case 1 0.0150 (0.0136, 0.0163); Case 2 0.0150 (0.0138, 0.0162).

* Study 3: True 0.015; Case 1 0.0150 (0.0137, 0.0162); Case 2 0.0149 (0.0133, 0.0167).

* Study 4: True 0.025; Case 1 0.0249 (0.0229, 0.0270); Case 2 0.0246 (0.0224, 0.0265).

* Study 5: True 0.01; Case 1 0.0092 (0.0060, 0.0131); Case 2 0.0068 (0.0016, 0.0115).

* Study 6: True 0.01; Case 1 0.0099 (0.0082, 0.0118); Case 2 0.0099 (0.0083, 0.0115).

* Study 7: True 0.018; Case 1 0.0175 (0.0145, 0.0214); Case 2 0.0180 (0.0152, 0.0209).

* Study 8: True 0.02; Case 1 0.0199 (0.0184, 0.0215); Case 2 0.0200 (0.0181, 0.0221).

* Study 9: True 0.012; Case 1 0.0118 (0.0088, 0.0155); Case 2 0.0117 (0.0092, 0.0147).

* Study 10: True 0.01; Case 1 0.0096 (0.0072, 0.0123); Case 2 0.0101 (0.0081, 0.0120).

* Study 11: True 0.024; Case 1 0.0240 (0.0218, 0.0261); Case 2 0.0240 (0.0221, 0.0263).

* Study 12: True 0.015; Case 1 0.0150 (0.0140, 0.0160); Case 2 0.0150 (0.0139, 0.0161).

* Study 13: True 0.01; Case 1 0.0098 (0.0084, 0.0115); Case 2 0.0101 (0.0086, 0.0117).

Navigate back to Table 10.
